# Ubc9 regulates the expression of MHC II in dendritic cells to enhance DSS-induced colitis by mediating RBPJ SUMOylation

**DOI:** 10.1038/s41419-023-06266-1

**Published:** 2023-11-13

**Authors:** Jing Zhang, Longmin Chen, Qianqian Xu, Yuan Zou, Fei Sun, Qing Zhou, Xi Luo, Yang Li, Cai Chen, Shu Zhang, Fei Xiong, Ping Yang, Shiwei Liu, Cong-Yi Wang

**Affiliations:** 1 Department of Respiratory and Critical Care Medicine, the Center for Biomedical Research, NHC Key Laboratory of Respiratory Diseases, Tongji Hospital Research Building, Tongji Hospital, Tongji Medical College, Huazhong University of Science and Technology, Wuhan, China; 2 Department of Rheumatology and Immunology, the Central Hospital of Wuhan, Tongji Medical College, Huazhong University of Science and Technology, Wuhan, China; 3 Department of Endocrinology, the Central Hospital of Wuhan, Tongji Medical College, Huazhong University of Science and Technology, Wuhan, China; 4 Shanxi Bethune Hospital, Shanxi Academy of Medical Science, Tongji Shanxi Hospital, Third Hospital of Shanxi Medical University, the Key Laboratory of Endocrine and Metabolic Diseases of Shanxi Province, Taiyuan, China

**Keywords:** Inflammatory bowel disease, Dendritic cells

## Abstract

SUMOylation is an evolutionary conserved regulatory mechanism, in which Ubc9 is the only E2 conjugating enzyme. Previous studies demonstrated that SUMOylation is involved in multiple biological processes, but its role in dendritic cells (DCs) remains to be fully addressed. Herein in this report, we found that DCs deficient in *Ubc9* protected mice from dextran sulfate sodium (DSS)-induced colitis, as evidenced by the ameliorated weight loss, colon length, and disrupted colon structure. Mechanistically, Ubc9 mediated SUMOylation of RBPJ, by which it stabilized RBPJ from ubiquitin-mediated degradation to enhance its transcriptional activity, while Ciita, a critical transcription factor, is a direct target downstream of RBPJ, which forms an enhanceosome complex to transcribe the expression of *MHC II* genes. Therefore, loss of *Ubc9* abolished RBPJ SUMOylation, which was coupled with reduced *Ciita* transcription, thereby attenuating the expression of MHC class II genes. As a consequence of defective MHC II expression, *Ubc9*^*-/-*^ DCs were featured by the impaired capability to process antigen and to prime effector CD4^+^ T cells, thereby protecting mice from DSS-induced colitis. Together, our results shed novel insight into the understanding of SUMOylation in the regulation of DC functions in pathological conditions.

## Introduction

Inflammatory bowel disease (IBD), which comprises Crohn’s disease (CD) and ulcerative colitis (UC), is a chronic inflammatory disorder of the gastrointestinal tract [1]. The pathogenesis of IBD is multifactorial, involving genetic, environmental, epithelial, microbial, and immune factors [2]. Particularly, the aberrant immune response of CD4^+^ T cells plays a key role in the disturbances of gut homeostasis, and infiltration of activated CD4^+^ T cells in the inflamed intestinal mucosa is considered as a characteristic feature of experimental murine colitis and human IBD [3, 4]. There is also feasible evidence supporting that CD4^+^ T cells are involved in the pathogenesis of IBD through interacting with other immunocytes or through upregulating the production of proinflammatory cytokines. Nevertheless, the underlying mechanisms by which CD4^+^ T cells are activated at the early stage of intestinal mucosal inflammation and participate in the development of IBD, have not been fully addressed.

Dendritic cells (DCs) are professional antigen-presenting cells (APCs) to bridge innate and adaptive immune response [5]. They are derived from hematopoietic stem cells accommodated in the bone marrow, and immature DCs are freshly generated to induce immune tolerance [6]. Distributed throughout nearly all the lymphoid and non-lymphoid tissues, DCs can be activated by ‘danger signals’ from both invading pathogens and injured host cells through pattern recognition receptors (PRRs) [7]. During the maturation process, DCs markedly upregulate the expression of major histocompatibility complex (MHC) class II, co-stimulatory molecules, and proinflammatory cytokines to prime T cell clonal expansion and differentiation to initiate adaptive immune response. Therefore, altered DC activation is closely related to various inflammatory and autoimmune diseases. Although the transcriptional circuitry that controls the development and function of DCs has been intensively investigated [8], the regulatory machineries of post-translational modifications (PTMs), particularly the role of Ubc9-mediated SUMOylation in this process, are yet to be fully elucidated.

SUMOylation is a highly transient and reversible PTM, characterized by the covalent attachment of the small ubiquitin-like modifier (SUMO) moiety to lysine residues within the target proteins [9]. Similar as the ubiquitination cascade, SUMOylation is carried out by the E1 activating enzyme, E2 conjugating enzyme, and E3 ligase, in which Ubc9 is the only E2 conjugating enzyme in mammalian cells, and therefore, deletion of *Ubc9* completely abolishes the SUMOylation function. Previously, we demonstrated that SUMOylation plays an important role to modulate immune tolerance by regulating the functionality of PDPK1 to maintain the stability of regulatory T (Treg) cells [10]. Similarly, loss of *Ubc9* attenuates macrophage M2 polarization, thereby exacerbating multiple-low dose streptozotocin-induced diabetes [11]. There is also evidence that SUMOylation restrains TLR-induced production of inflammatory cytokines and the expression of type I interferon (IFN) signature genes in DCs, in which SUMO operates from a distal enhancer of the gene encoding IFN-β (*Ifnb1*) to silence both basal and stimulus-induced activity of the *Ifnb1* promoter [12]. Therefore, the direct effect of SUMOylation on DC functions is yet to be fully addressed. To this end, a DC-specific *Ubc9* deficient mouse model was established in the current report to address the effect of SUMOylation on DC activation and functionality. It was noted that *Ubc9* deficiency abolished RBPJ (recombination signal binding protein for immunoglobulin kappa J region) SUMOylation, which affected its stability and transcriptional activity, thereby suppressing the expression of class II transactivator (Ciita), a master regulator of MHC class II genes. In consistent with these results, *Ubc9* deficient DCs manifested decreased expression of MHC class II and reduced capability to stimulate T cell responses, which protected mice from dextran sulfate sodium (DSS)-induced colitis along with a marked reduction of Th1 and Th17 cell accumulation in the mesenteric lymph nodes (MLNs) and colonic tissues. Together, those data provide a novel insight into the understanding how SUMOylation regulates DC functions relevant to the pathogenesis of colitis.

## Results

### Generation of a DC-specific *Ubc9* deficient mouse model

**Fig. 1:**
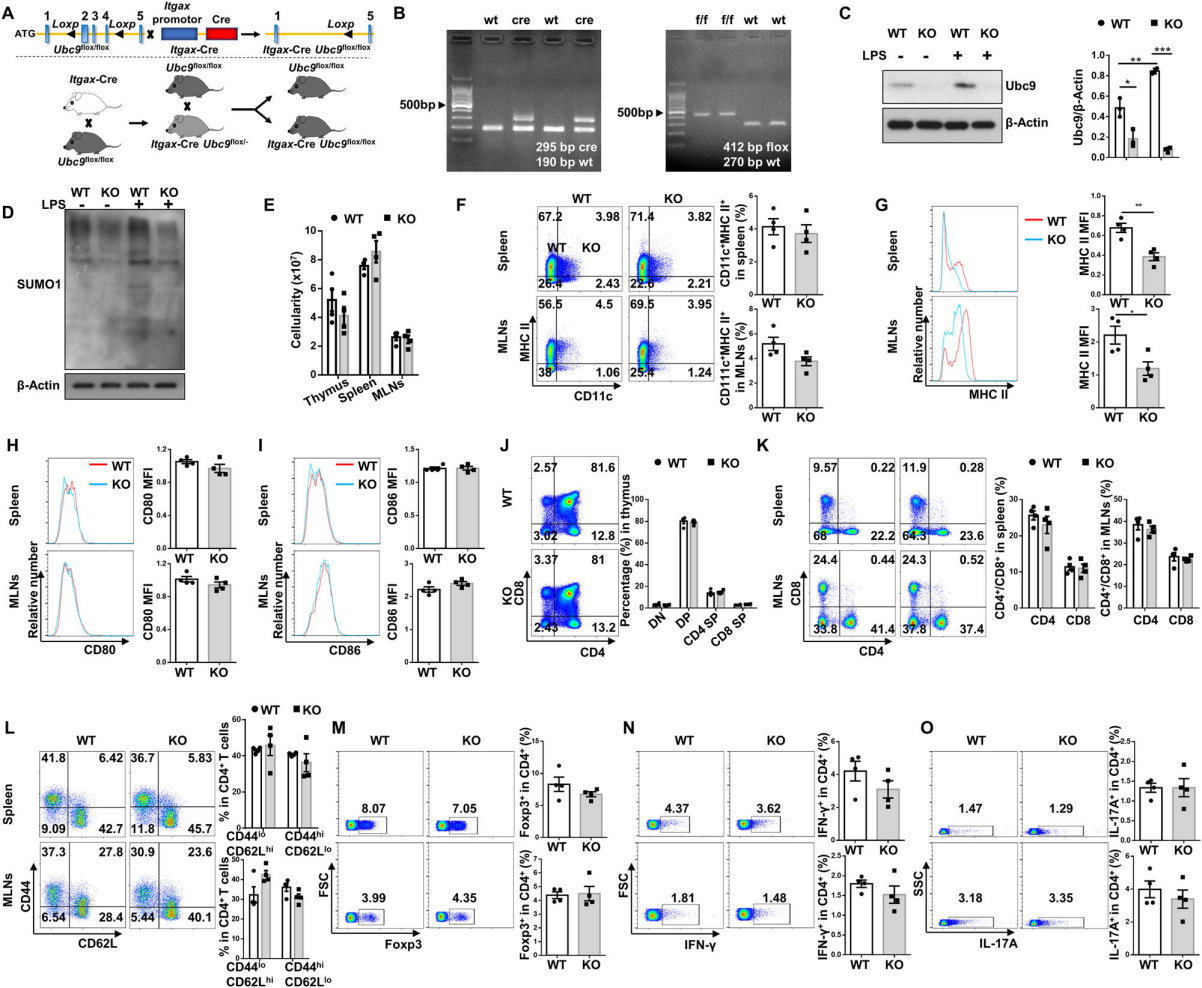
Establishment and characterization of a DC-specific *Ubc9* knockout mouse model. **A** *Ubc9*^flox/flox^ mice were generated by inserting two loxP sites in the same direction into intron sequences surrounding exons 2–4 of *Ubc9* based on CRISPR technology, which could generate a stop codon in exon 1 to produce a nonfunctional Ubc9 protein after Cre-mediated gene excision. *Ubc9*^flox/flox^ mice were then crossed with the *Itgax*-Cre transgenic mice to get the *Itgax*-Cre *Ubc9*^flox/flox^ mice and their littermates for following studies. **B** Genotyping results of the *Itgax*-Cre and flox alleles. **C**, **D** Western blot analysis of Ubc9 expression (**C**) and SUMOylated proteins (**D**) in BMDCs with or without LPS stimulation for 24 h. **E** Total cellularity of the thymus, spleen, and mesenteric lymph nodes (MLNs). **F** Representative FACS plots and frequencies of CD11c^+^MHC II^+^ cells in the spleen and MLNs of WT and KO mice. **G** MHC II expression in CD11c positive cells shown as median fluorescence intensity (MFI). **H**, **I** Flow cytometry analysis of CD80 (**H**) and CD86 (**I**) on DCs from spleen and MLNs. (**J**) Representative FACS plots and percentages of CD4 single positive, CD8 single positive, CD4/CD8 double positive, and CD4/CD8 double negative subsets in the thymus from WT and KO mice. **K** Representative FACS plots and frequencies of CD4^+^ and CD8^+^ T cells in the spleen and MLNs. **L** Representative FACS plots and percentages of CD44^high^CD62L^lo^ and CD44^lo^CD62L^high^ effector/naïve subpopulations gated on CD4^+^ T cells. **M**–**O** Representative FACS plots and proportions of Foxp3^+^ cells (**M**), IFN-γ^+^ cells (**N**), and IL-17A^+^ (**O**) cells within the CD4^+^ T cell population in the spleen and MLNs. *n* = 4 per group (**E**–**O**). Values are expressed as mean ± SEM. Significance was determined by one-way ANOVA in (**C**) and by unpaired Student’s *t* test in (**E**–**O**). *p < 0.05; **p < 0.01; ***p < 0.001.

We first crossed the *Ubc9*^f/f^ mice [13] with *Itgax*-Cre mice to generate a DC-specific *Ubc9* knockout mouse model (*Itgax*-Cre/*Ubc9*^f/f^ mice, hereinafter defined as KO mice), and their littermates (*Ubc9*^f/f^ mice, hereinafter denoted as WT mice) were served as controls (Fig. 1A). Genotyping of tail DNA confirmed *Ubc9* depletion as manifested by the presence of the *Cre* and *flox* alleles (Fig. 1B), which was further confirmed by the absence of Ubc9 protein in bone marrow-derived dendritic cells (BMDCs) (Fig. 1C). Interestingly, Ubc9 was remarkably upregulated in WT BMDCs upon LPS stimulation along with an increase of global protein SUMOylation levels (Fig. 1D), suggesting that Ubc9-mediated SUMOylation may play a critical role in DC functionality.

It was noted that the cellularity of thymus, spleen and MLNs was unaltered in 8-week-old KO mice under physiological condition (Fig. 1E). Similarly, the frequency of DCs in the spleen and MLNs from WT and KO mice did not show a significant difference (Fig. 1F). In sharp contrast, *Ubc9* deficient DCs manifested decreased expression of MHC class II (Fig. 1G), but not co-stimulatory molecules such as CD80 (Fig. 1H) and CD86 (Fig. 1I). Given the crucial role of DCs in priming T cells, we then examined T cell subpopulations. *Ubc9* deficiency did not affect thymocyte development (Fig. 1J) or peripheral T cell frequency (Fig. 1K). Although the KO mice exhibited a slightly higher proportion of CD44^lo^CD62L^hi^ naïve and lower proportion of CD44^hi^CD62L^lo^ effector T cells in the MLNs, they did not reach a statistical significance (Fig. 1L). Similarly, the proportions of Treg (Fig. 1M), Th1 (Fig. 1N), and Th17 (Fig. 1O) cells were comparable between two groups.

### DCs deficient in *Ubc9* protect mice from DSS-induce colitis

**Fig. 2:**
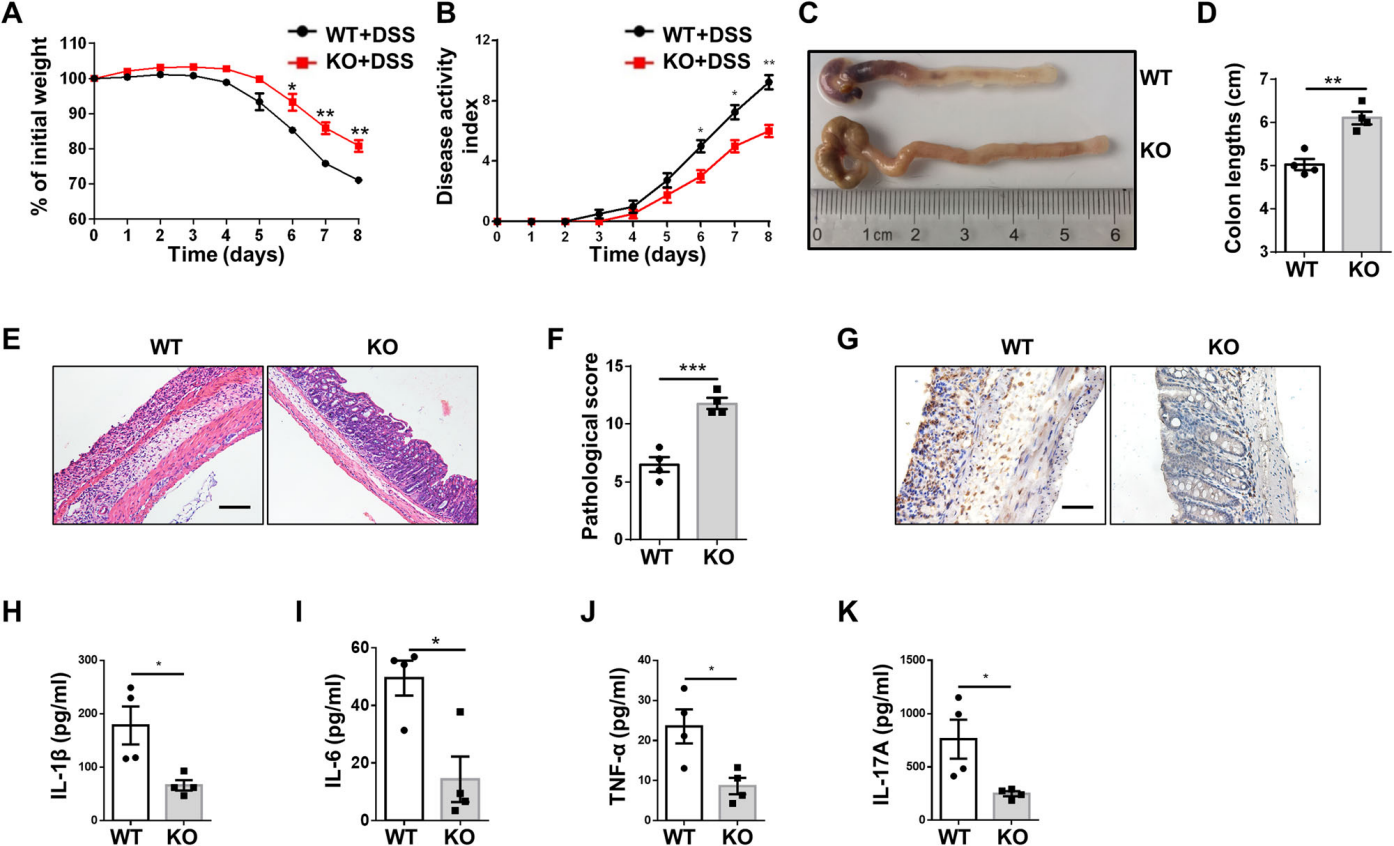
Mice with specific *Ubc9* deficiency in DCs are protected from DSS-induced colitis. **A** Body weight changes for WT and KO mice that challenged with DSS in drinking water. **B** The disease activity index (DAI) in mice after DSS administration. **C**, **D** Representative image of the colons (**C**) and graph showing colon lengths (**D**) on day 10 following DSS challenge. **E** Representative H&E staining of colons 10 days after DSS induction. **F** Graph summarizing histological severity score. **G** Representative results of immunohistochemical staining for MPO in sections of the colon. **H**–**K** Analysis of plasma IL-1β (**H**), IL-6 (**I**), TNF-α (**J**), and IL-17A (**K**) levels between DSS-challenged WT and KO mice (*n* = 4 for each group). Scale bars: 100 μm (**E**); 50 μm (**G**). Original magnification: ×200 (**E**); ×400 (**G**). Values are represented as mean ± SEM and unpaired Student’s *t* test was employed for statistical analysis. *p < 0.05; **p < 0.01; ***p < 0.001.

Next, we sought to investigate the effect of *Ubc9* deficiency under pathological conditions, and an IBD model was employed for the study. The KO mice and WT littermates were challenged with 3% DSS to induce colitis, respectively, which is a commonly used model to mimic human IBD. The KO mice developed less severe colitis as compared to that of WT controls, as evidenced by the less loss of body weight (Fig. 2A), lower disease activity index (DAI) (Fig. 2B), longer colons (Fig. 2C, D), and attenuated inflammatory infiltration along with lower severity of architectural damage in the colon (Fig. 2E, F). Immunohistochemical staining of colon sections further confirmed repressed inflammation as evidenced by the alleviated MPO levels (Fig. 2G). In consistent with those observations, a significant lower level of serum proinflammatory cytokines including IL-1β (Fig. 2H), IL-6 (Fig. 2I), TNF-α (Fig. 2J), and IL-17A (Fig. 2K) was detected in the KO mice. To explore whether the reduced levels of IL-6 and TNF-α originated from DCs or T cells, we further performed intracellular staining of IL-6 and TNF-α on DCs versus T cells. A significant decrease of IL-6 and TNF-α was observed in the MLN T cells of KO mice, but no significant difference was detected in DCs (Supplementary Fig. 1), indicating a pivotal contribution of T cells to the decreased serum IL-6 and TNF-α levels. Together, these results suggest that loss of *Ubc9* in DCs effectively attenuates DSS-induced colitis.

### DCs deficient in *Ubc9* exhibit repressed MHC class II expression along with lower capability for antigen processing

**Fig. 3:**
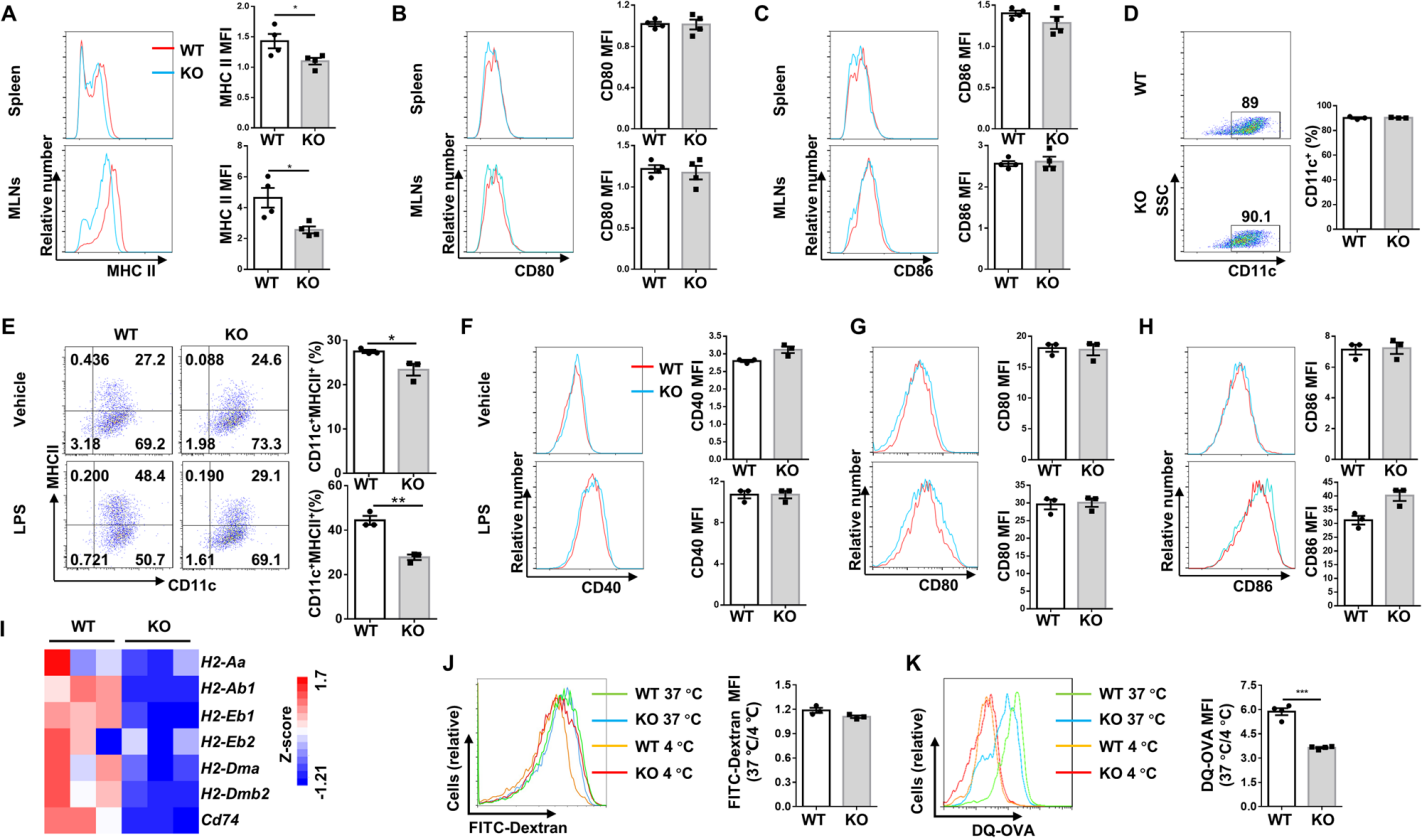
DCs deficient in *Ubc9* manifest repressed MHC class II expression along with lower antigen processing capability. **A**–**C** Single-cell suspension was prepared from mouse spleen and MLNs on day 10 of DSS induction, and subject to flow cytometry analysis of MHC class II (**A**), CD80 (**B**), and CD86 (**C**) expression in DCs. The MFI values are shown as bar graphs. *n* = 4 for each group. **D** Flow cytometry analysis of CD11c expression in BMDCs. **E** Frequencies of MHC II^+^ DCs from BMDC cultures of WT and KO mice, either left unstimulated or treated with LPS for 24 h. **F**–**H** FACS analysis of the expression of CD40 (**F**), CD80 (**G**), and CD86 (**H**) by WT and KO BMDCs treated with medium alone or LPS for 24 h. **I** mRNA expression of MHC II-related genes in LPS-stimulated WT and KO BMDCs. **J** Uptake of FITC-Dextran in BMDCs by flow cytometry analysis. **K** Antigen processing was determined by measurement of the fluorescence upon proteolytic degradation of the self-quenched conjugate DQ-OVA. Data are shown as mean ± SEM of 3 (**D**–**J**) or 4 (**K**) independent experiments. Statistical significance was accessed by unpaired Student’s *t* test. *p < 0.05; **p < 0.01; ***p < 0.001.

We first checked MHC class II and co-stimulatory molecule expressions in DCs of DSS-induced mice. *Ubc9* deficient DCs showed decreased expression of MHC class II in the spleen and MLNs after DSS challenge (Fig. 3A), while CD80 (Fig. 3B) and CD86 (Fig. 3C) expression did not show a significant difference. The production of CD11c^+^ BMDCs from WT and KO bone marrow were similar (Fig. 3D), suggesting that loss of *Ubc9* did not affect DC generation from precursor cells. Similar as the in vivo data (Fig. 1G), the KO BMDCs manifested a slightly lower MHC class II expression before LPS stimulation, but a much more significant reduction was noted following LPS induction (Fig. 3E). However, no significant difference was observed in terms of CD40 (Fig. 3F), CD80 (Fig. 3G) and CD86 (Fig. 3H) expression in LPS-stimulated KO BMDCs. Indeed, RT-PCR analysis confirmed significantly decreased mRNA levels for a set of MHC class II genes (Fig. 3I). These findings prompted us to examine the impact of *Ubc9* deficiency on DC antigen processing and presentation. Although the KO BMDCs displayed comparable capacity for dextran uptake as that of WT BMDCs (Fig. 3J), the mean fluorescence intensity (MFI), however, was significantly reduced in *Ubc9* deficient DCs pulsed with DQ-OVA (Fig. 3K), indicating an impaired capability for antigen processing.

### *Ubc9* deficiency decreases CD4^+^ effector T cells in the MLNs and colonic tissues of mice with onset of IBD

**Fig. 4:**
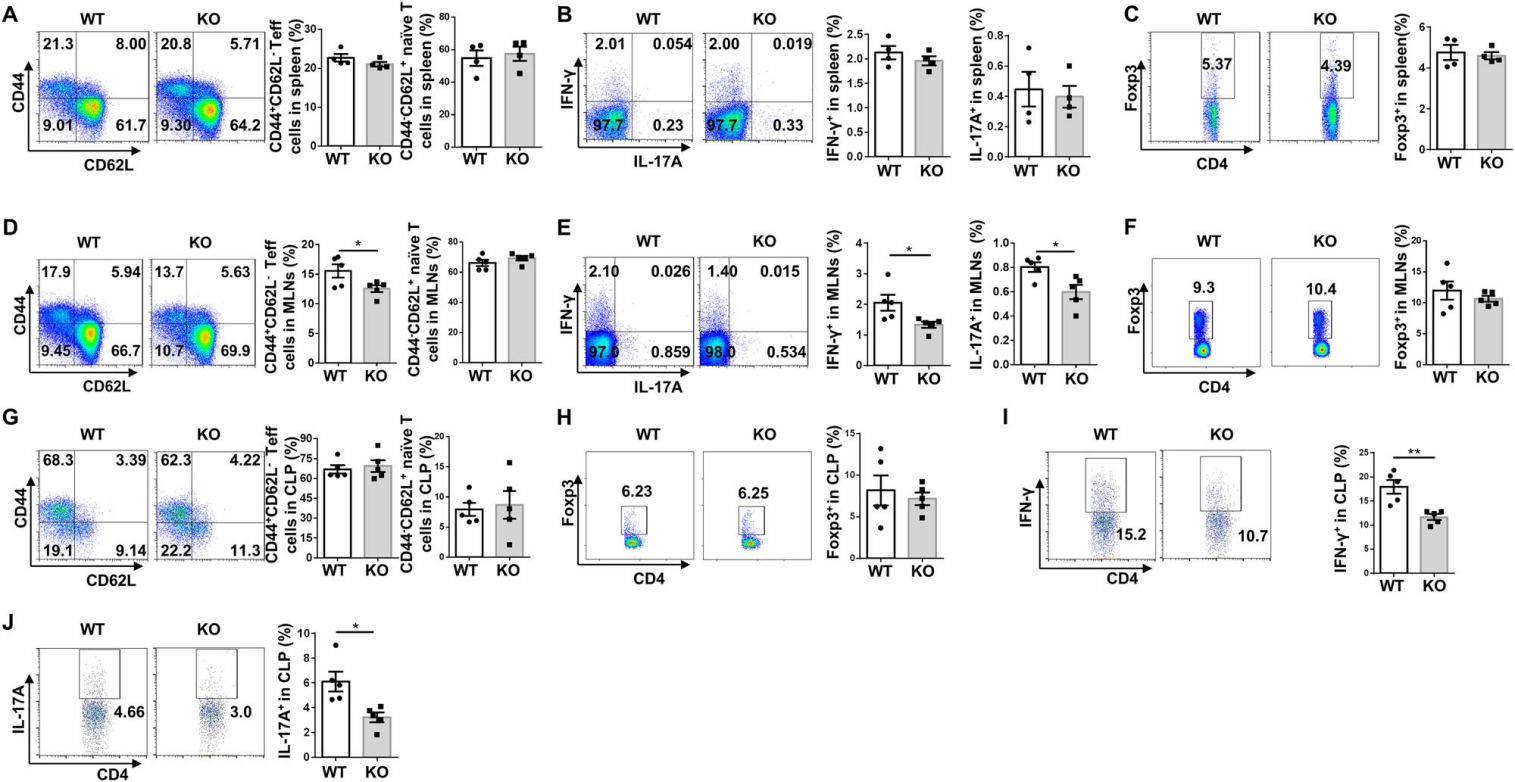
Loss of *Ubc9* decreases CD4^+^ effector T cells in the MLNs and colonic tissues of mice with IBD onset. Single-cell suspension was prepared from mouse spleen, MLNs, and colonic tissues on day 10 of DSS induction and subject to flow cytometry analysis. **A**–**C** Representative FACS plots and frequencies of CD44^high^CD62L^lo^ and CD44^lo^CD62L^high^ cells (**A**), IFN-γ^+^ and IL-17A^+^ cells (**B**), and Foxp3^+^ cells (**C**) gated on CD4^+^ splenocytes. *n* = 4 per group. **D**–**F** Representative FACS plots and percentages of CD44^high^CD62L^lo^ and CD44^lo^CD62L^high^ cells (**D**), IFN-γ^+^ and IL-17A^+^ cells (**E**), and Foxp3^+^ cells (**F**) within the CD4^+^ T cell population in the MLNs. *n* = 5 per group. (**G**–**J**) Representative FACS plots and proportions of CD44^high^CD62L^lo^ and CD44^lo^CD62L^high^ cells (**G**), Foxp3^+^ cells (**H**), IFN-γ^+^ cells (**I**), and IL-17A^+^ cells (**J**) in CD4^+^ T cells of colonic tissues. *n* = 5 per group. Data are expressed as mean ± SEM. Statistical difference was determined by unpaired Student’s *t* test. *p < 0.05; **p < 0.01.

Next, we checked CD4^+^ T cell subpopulations in the spleen, MLNs and colonic tissues at day 10 following DSS induction. In the spleen, the proportions of naïve and effector T cells (Fig. 4A), Th1, Th17 (Fig. 4B), and Treg (Fig. 4C) subsets were comparable between two groups of mice. However, less amount of effector T cells was noted in the MLNs of KO mice (Fig. 4D). Moreover, the KO mice displayed lower proportions of Th1 and Th17 cells in the MLNs (Fig. 4E), but without a perceptible difference in Treg cells (Fig. 4F). Although no significant difference in terms of the frequencies of naïve and effector T cells (Fig. 4G), as well as Treg cells (Fig. 4H), was noted in the colonic lamina propria, the proportions of colonic infiltrating Th1 (Fig. 4I) and Th17 (Fig. 4J) cells in KO mice, however, were dramatically reduced, as compared to those in WT mice following DSS induction. Collectively, our data support that *Ubc9* deficiency in DCs preferentially inhibits the generation of Th1 and Th17 cells, thereby ameliorating inflammatory response during the course of IBD development.

### Ubc9 mediates SUMOylation of RBPJ

**Fig. 5:**
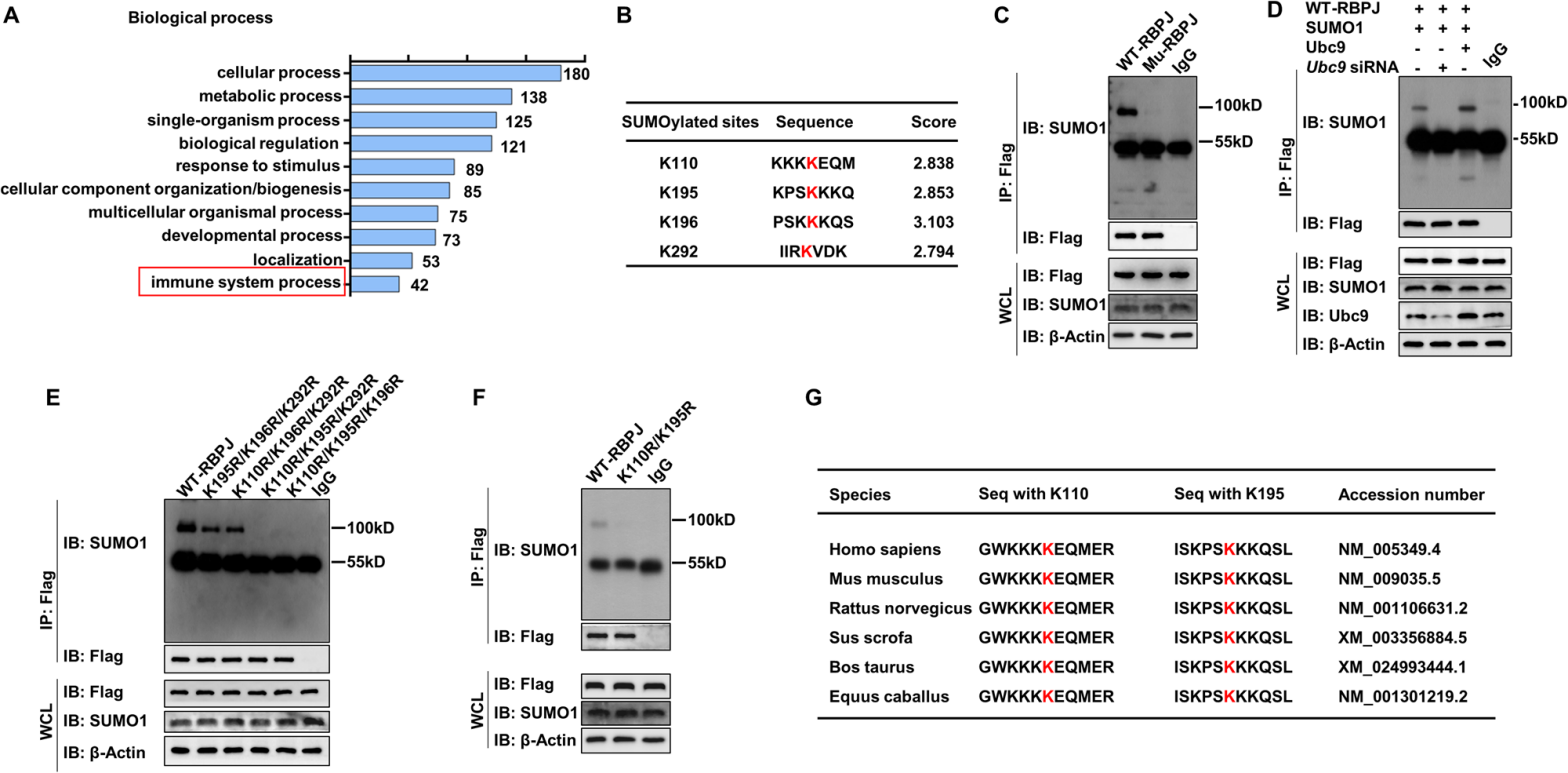
Ubc9 mediates SUMOylation of RBPJ. **A** A cut-off was set up (LPS-stimulated group versus unstimulated group) as fold changes for quantitative analysis. GO enrichment-based clustering was applied to analyze the biological process of potential SUMOylated proteins. **B** Predicted SUMOylation sites in RBPJ. **C** Assay for the SUMOylation of RBPJ in HEK293T cells transfected with plasmids encoding Flag-tagged WT RBPJ or lysine-to-arginine RBPJ mutant, together with plasmid encoding SUMO1. **D** SUMOylation analysis using HEK293T cells transfected with the indicated vectors. **E** RBPJ SUMOylation assay using HEK293T cells co-transfected with WT RBPJ or its triple mutant variants and SUMO1. **F** WT RBPJ or K110R/K195R mutant RBPJ were individually transfected with SUMO1 into HEK293T cells for RBPJ SUMOylation assay. **G** Sequence alignment surrounding K110 and K195 in RBPJ from the indicated species. Data are representative of three independent experiments in (**C**–**F**).

To dissect the mechanisms by which *Ubc9* deficiency represses MHC II expression in DCs, we conducted comparative proteomic analyses of SUMOylated substrates in DCs before/after LPS stimulation. Gene ontology (GO) analysis revealed that the SUMOylated proteins were enriched in immune system process (Fig. 5A), among which RBPJ was the most critical substrate relevant to MHC II expression, which was then selected for the subsequent studies. Bioinformatic analysis predicted lysine (K) residues at positions 110, 195, 196, and 292 as the possible SUMOylation sites in RBPJ (Fig. 5B). To confirm that RBPJ is a substrate for SUMOylation, plasmids expressing Flag-tagged WT or mutant (MU) RBPJ (the predicted K residues were mutated to arginine) were constructed. The plasmids were next co-transfected with SUMO1 into HEK293T cells, which were then subjected to co-immunoprecipitation (co-IP) using the Flag antibody, and the resulting products were probed by SUMO1. Indeed, the SUMOylated band with higher molecular weight than that of RBPJ was detected in WT plasmid transfected cells (Fig. 5C), indicating that RBPJ could be SUMOylated. Next, to investigate whether the SUMOylation of RBPJ was mediated by Ubc9, we knocked down *Ubc9* using siRNA or overexpressed it in HEK293T cells transfected with SUMO1 and WT RBPJ plasmids, followed by SUMOylation analysis. The result showed that knockdown of *Ubc9* almost abolished the SUMOylation band, while *Ubc9* overexpression significantly promoted RBPJ SUMOylation (Fig. 5D), suggesting that Ubc9 mediates RBPJ SUMOylation.

To identify the exact lysine residues for SUMOylation, we constructed a combination of triple-mutated plasmids (K195R/K196R/K292R, K110R/K196R/K292R, K110R/K195R/K292R, and K110R/K195R/K196R). It was noted that the SUMOylation levels of cells transfected with either K195R/K196R/K292R or K110R/K196R/K292R RBPJ plasmid were significantly reduced as compared to that of WT PBPJ transfected cells, while the SUMOylated band was completely absent in cells transfected with either K110R/K195R/K292R or K110R/K195R/K196R mutants (Fig. 5E), indicating that K110 and K195 are likely the SUMOylation sites. Indeed, once the cells transfected K110R/K195R mutant, the SUMOylated RBPJ could not be detected (Fig. 5F). Sequence alignment of RBPJ among species showed that K110 and K195 and their surrounding amino acids were evolutionarily conserved (Fig. 5G), supporting that SUMOylation of K110 and K195 of RBPJ may play a critical role to regulate DC function.

### SUMOylation of RBPJ regulates MHC II expression by enhancing *Ciita* transcription

**Fig. 6:**
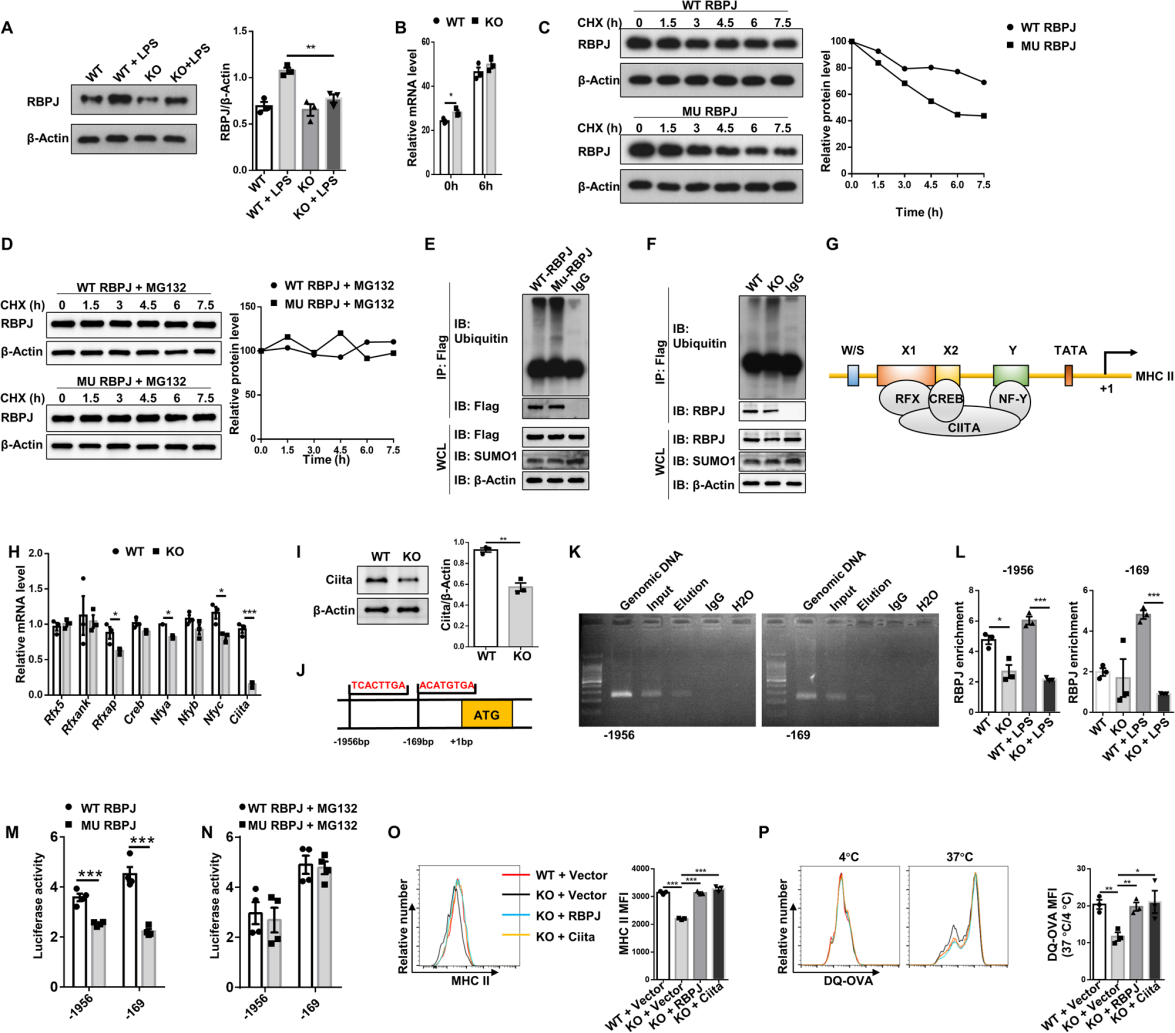
Ubc9-mediated SUMOylation stabilizes RBPJ from ubiquitin-mediated degradation to enhance *Ciita* transcription. **A** Western blot analysis of RBPJ expression in BMDCs before and after LPS stimulation. **B** RT-PCR results for *Rbpj* expression in BMDCs treated with vehicle or LPS for 6 h. **C**, **D** HEK293T cells were transfected with plasmids expressing Flag-tagged RBPJ in the presence of CHX for indicated time, and Western blot analysis was conducted to analyze the half-life of RBPJ. Proteasome inhibitor MG132 was added in (**D**). **E** Results of an ubiquitination assay. The Flag immunoprecipitates were probed with an ubiquitin antibody for analysis of RBPJ ubiquitination. **F** RBPJ ubiquitination assay using WT and KO BMDCs. **G** A model depicting the transcriptional regulation of MHC class II genes. **H** RT-PCR analysis of relative mRNA levels of *MHC II* regulatory factors in BMDCs at stimulated state. **I** Western blot analysis of Ciita expression in BMDCs after LPS stimulation. **J** The predicted RBPJ binding sites within the *Ciita* promoter. **K** ChIP-PCR results for the analysis of RBPJ binding capability to the *Ciita* promoter. **L** ChIP-qPCR was performed for RBPJ in the *Ciita* promoter in WT and KO BMDCs treated with or without LPS. **M** Relative luciferase activity in HEK293T cells co-transfected with WT or MU RBPJ and SUMO1. **N** Dual-luciferase reporter assay performed as in (**M**) in the presence of MG132. **O**, **P** BMDCs were transduced with vector, WT RBPJ or Ciita adenovirus as indicated. Results for flow cytometry analysis of MHC class II expression (**O**) and antigen processing capability (**P**). All experiments were repeated independently 3 times. Values were expressed as mean ± SEM. Statistical significance was analyzed by one-way ANOVA in (**O** and **P**) and by unpaired Student’s *t* test in other figure parts. *p < 0.05; **p < 0.01; ***p < 0.001.

To address the impact of SUMOylation on RBPJ, we first examined RBPJ expression in DCs. RBPJ is highly expressed in BMDCs (Fig. 6A, lanes 1 and 3). Moreover, LPS potently induced RBPJ expression, which was significantly attenuated in *Ubc9* deficient DCs (Fig. 6A, lanes 2 and 4). As the mRNA abundance of *Rbpj* was similar between WT and KO BMDCs upon LPS stimulation (Fig. 6B), we thus assumed that SUMOylation may stabilize RBPJ from degradation. To address this question, HEK293T cells were transfected with WT or MU plasmids, followed by cycloheximide (CHX) treatment to inhibit protein synthesis. Indeed, the half-life of mutant RBPJ was significantly shorter in comparison to that of WT RBPJ protein (Fig. 6C), and this defect can be rescued by MG132 treatment (Fig. 6D). We then checked RBPJ ubiquitination in above transfected HEK293T cells. Indeed, the MU plasmid transfected cells displayed a higher level of the polyubiquitinated RBPJ than that of WT plasmid transfected cells in the presence of MG132 (Fig. 6E), supporting that SUMOylation protects RBPJ from proteasome-mediated degradation. In consistent with the results in HEK293T cells, increased ubiquitination level of RBPJ was also detected in *Ubc9*^-/-^ DCs (Fig. 6F).

Given that the transcription of MHC II genes is controlled by a group of ubiquitously expressed factors including regulatory factor X (RFX), cAMP-responsive element binding protein (CREB1), and nuclear factor Y (NFY), and all of which act in concert with the MHC II transactivator Ciita (Fig. 6G) [14], we thus conducted comparative analysis of these transcription regulators. Interestingly, *Ciita* was identified to be the most significant one downregulated in *Ubc9* deficient BMDCs (Fig. 6H), and Western blot analysis of Ciita confirmed a significantly lower protein expression level in KO BMDCs (Fig. 6I). To address whether RBPJ mediates *Ciita* transcription which is regulated by SUMOylation, we conducted in silico analysis and characterized two RBPJ binding sites within the *Ciita* promoter region (Fig. 6J). Indeed, chromatin immunoprecipitation (ChIP)-PCR verified that RBPJ binds to the regions flanking the above indicated putative sites (Fig. 6K). Furthermore, less amount of RBPJ was recruited to the *Ciita* promoter in KO BMDCs than that in WT BMDCs, especially in the presence of LPS (Fig. 6L). Consistently, luciferase reporter assay confirmed that the transcriptional activity of RBPJ was attenuated in cells transfected with MU plasmid than those transfected with WT plasmid (Fig. 6M). Furthermore, HEK293T cells transfected with WT or MU plasmid exhibited comparable luciferase activity in the presence of MG132, indicating that the attenuated transcriptional activity of MU RBPJ was caused by its enhanced degradation rather than by its reduced binding ability to the *Ciita* promoter (Fig. 6N).

Since repressed MHC class II expression along with lower capability for antigen processing were observed in KO DCs, we employed DCs for the rescue assays. Indeed, reintroduction of RBPJ or Ciita restored the deficit in *Ubc9*^-/-^ DCs (Fig. 6O, P). Collectively, those data support that Ubc9-mediated SUMOylation stabilizes RBPJ from ubiquitin-mediated degradation to enhance *Ciita* transcription, which then transcribes MHC II expression in DCs.

### DCs deficient in *Ubc9* manifest reduced capacity to prime T cells

**Fig. 7:**
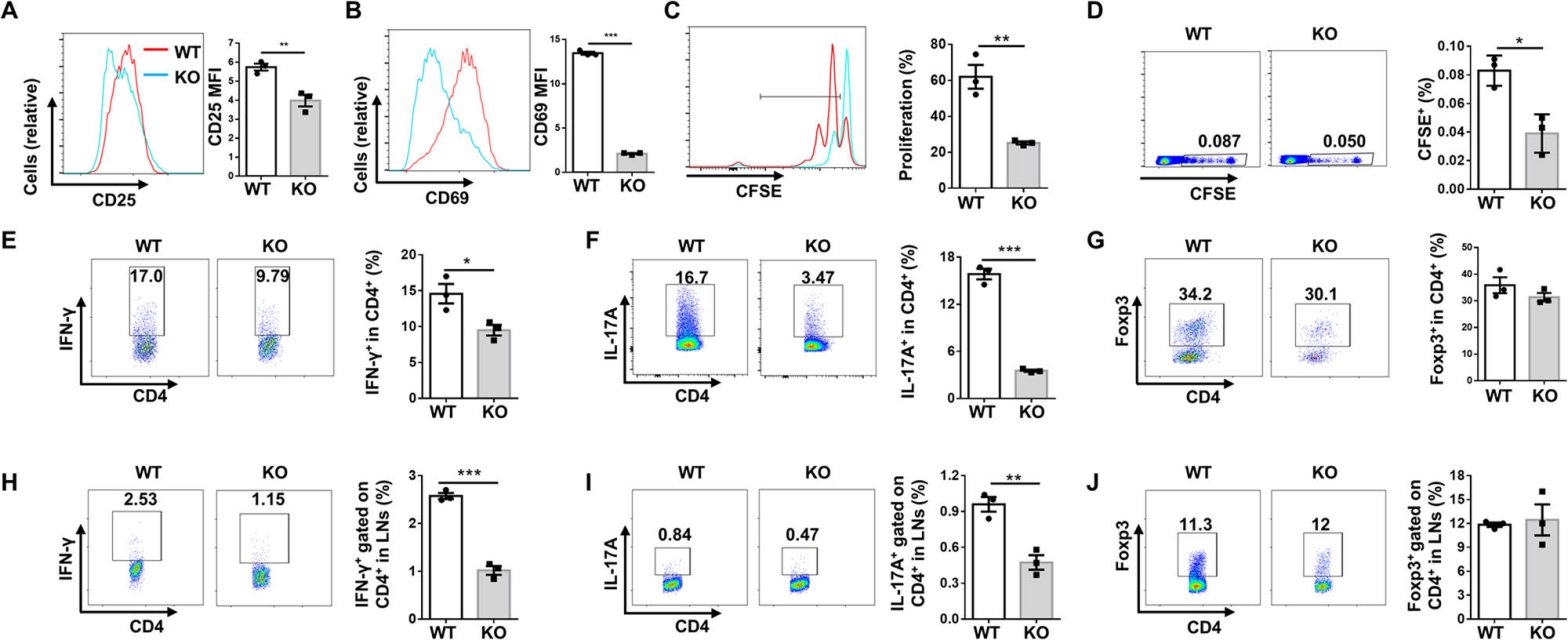
DCs deficient in *Ubc9* manifest impaired capability to prime CD4 T cells. **A**, **B** Flow cytometry analysis of T cell activation markers CD25 (**A**) and CD69 (**B**) on CD4^+^ OT-II T cells 24 h after co-culture with OVA_(323–339)_ peptide-pulsed BMDCs. **C** Proliferation of CFSE-labeled OT-II CD4^+^ T cells co-cultured with OVA peptide-pulsed DCs. **D** In vivo proliferation of OT-II CD4^+^ T cells in recipient mice immunized with OVA peptide-pulsed WT or KO BMDCs. **E**–**G** Proportions of IFN-γ^+^ cells (**E**), IL-17A^+^ cells (**F**), and Foxp3^+^ cells (**G**) after co-culture of OT-II naive CD4^+^ T cells with OVA peptide-pulsed BMDCs under Th1, Th17, and Treg conditions for 3 days. **H**–**J** In vivo polarization of OT-II naive CD4^+^ T cells to Th1 (**H**), Th17 (**I**), and Treg (**J**) cells in recipients immunized with OVA peptide-pulsed WT or KO DCs. Results are representative of 3 independent replications (**A**–**C** and **E**–**G**). *N* = 3 for each group in figures (**D**) and (**H**–**J**). Data are presented as mean ± SEM, and unpaired Student’s *t* test was used for data analysis. *p < 0.05; **p < 0.01; ***p < 0.001.

Finally, we checked the impact of *Ubc9* deficiency on the capability of DCs to prime CD4^+^ T cells. For this purpose, OT-II transgenic mice were employed for the study. As expected, OVA_(323–339)_-pulsed BMDCs from KO mice were less capable of inducing the expression of activation markers CD25 (Fig. 7A) and CD69 (Fig. 7B) on OT-II T cells, along with a reduced capacity to initiate OT-II T cell proliferation (Fig. 7C). Moreover, KO DCs manifested markedly decreased capability to prime OVA peptide-specific CD4^+^ T cells in the recipient mice, as evidenced by the significantly attenuated proliferation (Fig. 7D). To determine whether *Ubc9* deficiency influences T cell polarization program, we cultured OVA-pulsed WT and KO DCs with naive OT-II T cells under Th1, Th17, and Treg conditions, respectively. Compared to WT DCs, KO DCs induced lower percentages of Th1 (Fig. 7E) and Th17 (Fig. 7F) cells, but no discernable difference was detected in terms of the induction of Treg cells (Fig. 7G). Next, we adoptively transferred naive OT II CD4^+^ T cells into the recipient mice, followed by immunization with OVA-pulsed WT or KO DCs one day later, and similar results were noted (Fig. 7H–J). Altogether, our data support that Ubc9 plays an essential role in DCs to prime CD4^+^ T cells.

## Discussion

DCs represent a major type of innate immune cells to perceive tissue damage and pathogen invasion, and excess activation of T cells stimulated by mature DCs is closely associated with various pathological conditions. Previous studies have demonstrated convincing evidence that manipulation of DC function is a promising strategy to attenuate the severity of several inflammatory and autoimmune diseases [15–17]. Therefore, a number of clinical trials were carried out in colitis, arthritis, and multiple sclerosis by targeting DCs [18–21]. Presentation of antigen-derived peptides by MHC class II operates at the epicenter of immunity, connecting phagocytosis and microbial clearance with T cell activation and differentiation. Genome-wide association study (GWAS) identified multiple human leukocyte antigen (HLA) alleles confer risk to IBD susceptibility [22, 23]. However, the detailed regulatory mechanisms underlying MHC II expression in APCs, particularly in DCs, are yet to be fully addressed. In this report, we demonstrated an essential role of Ubc9-mediated SUMOylation in regulating MHC II expression in DCs, by which SUMOylation enhances the capability of DCs to prime T cell response predisposing to the development and progression of DSS-induced colitis.

Of note, comparative analysis of serum inflammatory cytokines revealed lower IL-6 and TNF-α in the KO mice 10 days after DSS induction. As adaptive immune response had been initiated at current stage, we conducted intracellular staining of IL-6 and TNF-α on DCs versus T cells to explore the source of reduced circulating IL-6 and TNF-α. A significant decrease of IL-6 and TNF-α was only observed in the MLN T cells of KO mice, but no significant difference was detected in DCs, implying that T cells are the primary contributor to the decreased IL-6 and TNF-α levels. This observation is in fact consistent with the reduced capacity of *Ubc9*^-/-^ DCs to prime T cells coupled with less severity of colitis.

To get insight into the mechanisms by which Ubc9-mediated SUMOylation promotes MHC II expression in DCs, we comparatively analyzed the endogenous SUMOylated substrates in DCs before/after LPS stimulation by MS, and RBPJ was identified as the most significant one following LPS stimulation. As a transcriptional factor, RBPJ is a key nuclear mediator of the canonical Notch pathway and involved in the development and function of a diversity of innate and adaptive immune cells [24]. There is feasible evidence that RBPJ plays an essential for DCs to evoke efficient anti-tumor immune response through affecting series of processes including maturation, migration, antigen presentation, and T cell activation [25]. For example, deletion of RBPJ in DCs compromises TLR-mediated DC activation [6, 26] along with reduced capability to stimulate T cell proliferation [25]. We thus embarked on RBPJ and confirmed that it is a substrate for Ubc9-mediated SUMOylation. Specifically, we demonstrated that K110 and K195 are the two lysine residues subjected to Ubc9-mediated SUMOylation. To investigate the effect of SUMOylation on RBPJ function, we generated a MU plasmid in which the SUMOylated lysine residues were replaced by arginine. Subsequent studies provided experimental evidence that SUMOylation stabilizes RBPJ from ubiquitin-mediated degradation, thereby enhancing its transcriptional activity.

Generally, MHC class II is constitutively expressed in DCs in the steady-state condition but markedly enhanced during DC activation and maturation. The expression of MHC class II is exquisitely controlled at the transcriptional levels [27]. The promoters of *MHC II* and related genes are featured by the presence of a highly conserved W/SXY module. The X1 box is recognized by RFX, a trimeric complex composed of RFX5, RFXANK, and RFXAP. The X2 box is bound by X2BP, a complex that contains CREB. Lastly, the trimeric NF-Y complex, made up of NF-YA, NF-YB, and NF-YC, binds to the Y box [28]. All of these factors bind cooperatively to the cis-regulatory elements of *MHC II* promoters to form an enhanceosome complex, which serves as a landing pad for Ciita. Our current results showed that *Ciita* rather than genes encoding for other transcription regulators was the mostly affected in *Ubc9* deficient DCs, and it is therefore, plausible to assume that the reduction of *Ciita* resulted from *Ubc9* deficiency contributes to the decline of the expression of MHC II genes. In support of this assumption, *CIITA* variants were found to be associated with IBD susceptibility in Asians [29].

The next key question is whether RBPJ transcribes *Ciita* expression and whether this process is regulated by Ubc9-mediated SUMOylation. In silico analysis of *Ciita* promoter characterized two potential RBPJ binding motifs located at positions −1956 and −169 bp (start codon as +1). ChIP assay was then performed and demonstrated that RBPJ bound to these two sites. Moreover, transfection of WT *Rbpj* was found to potently drive the *Ciita* reporter gene expression. In sharp contrast, analysis of the luminescence intensity revealed that the expression of the *Ciita* reporter gene was substantially reduced once the Mu *Rbpj* plasmid was transfected, supporting that SUMOylation of RBPJ enhances *Ciita* transcription to regulate MHC II expression in DCs.

In summary, our studies demonstrated that Ubc9 plays a critical role to regulate DC activation by enhancing MHC II expression, thereby predisposing to the initiation and progression of DSS-induced colitis. Mechanistically, Ubc9 mediates SUMOylation of RBPJ, by which it stabilizes RBPJ from ubiquitin-mediated degradation to enhance its transcriptional activity, while Ciita, a critical transcription factor, is a direct target downstream of RBPJ, which forms an enhanceosome complex to transcribe the expression of *MHC II* genes. Therefore, loss of *Ubc9* abolishes RBPJ SUMOylation, which is coupled with reduced *Ciita* transcription, thereby attenuating the expression of MHC class II genes. As a consequence of defective MHC II expression, the KO DCs were featured by the impaired capability to process antigen and to prime effector CD4^+^ T cells, thereby protecting mice from DSS-induced colitis. Together, our results shed novel insight into the understanding of SUMOylation in the regulation of DC functions in pathological conditions.

## Materials and methods

### Mice

The *Ubc9*^flox/flox^ (*Ubc9*^f/f^) mice were generated as described previously [13]. The *Itgax*-Cre transgenic mice and OT-II TCR transgenic mice were purchased from the Jackson’s Laboratory (Bar Harbor, ME, USA). *Itgax*-Cre *Ubc9*^f/f^ mice were generated by crossing the *Itgax*-Cre mice with *Ubc9*^f/f^ mice for specific deletion of *Ubc9* in DCs, and their *Ubc9*^f/f^ littermates were used as controls. C57BL/6 mice were purchased from Beijing HFK Bioscience (Beijing, China). All mice were bred in a specific pathogen-free (SPF) facility at the Tongji Hospital Animal Center with a 12-h light/12-h dark cycle. All protocols for animal studies were approved by the Tongji Hospital Animal Care and Use Committee (TJH-202005007) in accordance with the National Institutes of Health (NIH) guidelines. No randomization or blinding was used in animal studies.

### Western blot analysis and RT-PCR

Cells were lysed in RIPA lysis buffer (P0013B; Beyotime, Shanghai, China) containing protease inhibitors (Roche, IN, USA). Equal amounts of lysates were separated with SDS-PAGE and then transferred onto polyvinylidene difluoride membranes (Bio-Rad Laboratories, Hercules, Calif). After blocking with 5% nonfat milk for 1 h, the membrane was incubated with primary antibodies overnight at 4 °C, respectively. Antibody against DYKDDDDK Tag (2368S) was obtained from the Cell Signaling Technology (Danvers, MA, USA); antibody against Ciita (A16401) was ordered from Abclonal (Wuhan, China); antibodies against SUMO1 (10329-1-AP) and β-Actin (66009-1-Ig) were originated from Proteintech (Wuhan, China); and antibodies against Ubc9 (sc-271057) and RBPJK (ab25949) were purchased from Santa Cruz Biotechnology (Santa Cruz, CA, USA) and Abcam (Cambridge, MA, USA), respectively. After incubation for 1 h at room temperature with HRP-conjugated secondary antibodies, signals were visualized by enhanced ECL (E412-01; Vazyme, Nanjing, China) and were exposed on the ChemiDoc XRS+ system (Bio-Rad) or to X-ray film. Densitometry analysis was performed using the Image J 1.46r software.

Total RNA was extracted from DCs with the RNAiso Plus (9109; TaKaRa, Japan), and reverse transcription was conducted using a RevertAid First Strand cDNA Synthesis Kit (K1622; Thermo Fisher, San Francisco, CA, USA) following the manufacturer’s instructions. RT-PCR was then performed using the Hieff qPCR SYBR Green Master Mix (11203ES03; Yeasen, Shanghai, China) on an ABI prism 7500 Sequence Detection System (Applied Biosystems, CA, USA). The relative expression levels for each target gene were calculated with the *2*^*−ΔΔCt*^ method and normalized by *Actb*. Primer sequences for all examined genes are listed in Supplementary Table 1.

### Flow cytometry analysis

Single-cell suspension was prepared from mouse spleen, LNs, and colonic tissues or recovered from cell cultures. Cell surface markers were stained in PBS containing 1% BSA with relevant antibodies at 4 °C for 30 min. Intracellular staining was performed according to the manufacturer’s instructions for the Transcription Factor Buffer Set (562574; BD Biosciences, San Diego, CA, USA) with indicated antibodies. For intracellular cytokine staining, cells were first stimulated with Cell Activation Cocktail (with Brefeldin A) (423303; Biolegend, San Diego, CA, USA) for 4–6 h. The following antibodies were used for the studies: FITC anti-mouse CD11c (117306), Pacific Blue anti-mouse I-A/I-E (107620), PE/Cy7 anti-mouse CD86 (105014), APC anti-mouse CD40 (124612), FITC anti-mouse CD4 (100406), PerCP anti-mouse CD8a (100731), APC anti-mouse CD62L (104412), PE anti-mouse/human CD44 (103008), PE anti-mouse CD25 (113704), APC anti-mouse CD69 (104514), PE/Cy7 anti-mouse IFN-γ (505826), Brilliant Violet 421 anti-mouse IL-17A (506926), Alexa Fluor 647 anti-mouse/rat/human FOXP3 (320014), Brilliant Violet 421 anti-mouse CD3ε (100336), PE/Cy7 anti-mouse CD11c (117318), PE anti-mouse IL-6 (504503), and APC anti-mouse TNF-α (506307) from Biolegend (San Diego, CA, USA); and PE hamster anti-mouse CD80 (553769) from BD Biosciences (San Diego, CA, USA). All flow samples were acquired using a MACSQuant (Miltenyi Biotec, Auburn, CA, USA). Data were analyzed with FlowJo software (v10.5.3).

### DSS-induced colitis

Acute DSS-induced colitis was induced using established techniques [30]. Briefly, 8–10-week-old male mice were fed with 3% DSS (160110; MP Biomedicals, San Francisco, CA, USA) in drinking water for 7 days followed by 3 days of normal drinking water. During the course of experiment, diarrhea, weight change, and rectal bleeding were monitored daily, and disease activity index (DAI) was assessed for each animal for severity of colitis as previously described [31]. All mice were sacrificed at day 10, and the spleen, MLNs, and colonic tissues were collected for further analysis.

### Histological and immunohistochemical analysis

The colonic tissues were fixed in fresh 4% paraformaldehyde overnight, embedded in paraffin, and sectioned for staining with hematoxylin and eosin (H&E). The severity of colon inflammation was assessed in a blinded fashion by two pathologists using the previously described scoring system [32]. For immunohistochemical analysis, the sections (4 μm) were probed with antibody against MPO (GB12224; Servicebio, Wuhan, China), followed by counterstained with Harris’ hematoxylin as reported [33].

### Antigen uptake and processing assays

To assess the ability of DCs to uptake antigens, 5 × 10^5^ cells were suspended in 100 μl of culture medium and incubated with FITC-dextran (m.w. 70,000, D1951; Molecular Probes, Eugene, OR) for 30 min at 4 °C or 37 °C. Antigen uptake was terminated by rapid cooling of the cells on ice followed by washes with ice-cold PBS. Cells were then subjected to flow cytometry analysis.

Antigen processing was determined as previously reported [34]. Briefly, 5 × 10^5^ DCs were incubated at 4 °C or 37 °C with DQ-OVA (Molecular Probes, Eugene, OR), which was conjugated with a self-quenched fluorophore derivative that exhibits bright green fluorescence upon proteolytic degradation. After 30 min, the cells were washed and analyzed by flow cytometry.

### DC-T cell co-culture

Naive T cells were enriched from the spleen of OT-II mice with a Naive CD4^+^ T Cell Isolation Kit for mouse (130-104-453; Miltenyi Biotec, Auburn, CA, USA) and labeled with CFSE. LPS-activated DCs was pulsed with OVA_323–339_ (O1641; Sigma-Aldrich, St. Louis, MO, USA) for 2 h and then co-cultured with OVA-specific transgenic CD4^+^ OT-II T cells at a ratio of 1:5. T cell proliferation was measured after 3 days by flow cytometry as the dilution of CFSE. For polarization assay, the following lineage-commitment cocktails were added: Th1, 10 ng/ml IL-2 (575404; Biolegend, San Diego, CA, USA) + 10 ng/ml IL-12 (577004; Biolegend); Th17, 50 ng/ml IL-6 (575704; Biolegend) + 2 ng/ml TGF-β (100-21; PeproTech, Rocky Hill, Connecticut, USA) + 10 μg/ml anti-IFN-γ (505848; Biolegend) + 10 μg/ml anti-IL-4 (504102; Biolegend); Treg, 10 ng/ml IL-2 + 5 ng/ml TGF-β. Three days later, the cells were harvested for flow cytometry analysis.

### In vivo T cell proliferation assay

CFSE-labeled naive OT-II CD4^+^ T cells were injected i.v. into recipient mice (2 × 10^6^ cells/mouse). One day later, BMDCs from WT or KO mice pulse with OVA_(323–339)_ for 2 h were transferred subcutaneously into the hind footpads of the recipients (6 × 10^5^ cells/mouse). After 3 days, popliteal lymph nodes were collected, and the percentage of CFSE^+^ cells was analyzed.

### SUMOylation analysis

HEK293T cells (ATCC, free for mycoplasma and authenticated by STR profiling) were transfected with plasmids containing SUMO1 and Flag tagged RBPJ. The cells were harvested 48 h after transfection, washed with ice-cold PBS, and lysed on ice for 30 min in IP lysis buffer (P0013; Beyotime, Shanghai, China) containing protease inhibitors, phosphatase inhibitors (G2007; Servicebio, Wuhan, China), 20 mM N-ethylmaleimide (Sigma, St Louis, MO, USA), and 1 mM PMSF (G2008; Servicebio, Wuhan, China). Cell lysates were cleared by centrifugation, and supernatants were incubated with 5 µg of indicated antibodies overnight and immunoprecipitated for an additional 4 h at 4 °C with Dynabeads Protein G (10004D; Invitrogen, Carlsbad, CA). The samples were next used for Western blot analysis with the appropriate antibodies.

### Chromatin immunoprecipitation (ChIP) assay

ChIP assay was performed using the ChIP Assay Kit (P2078; Beyotime, Shanghai, China) as previously reported [35]. In brief, 2 × 10^6^ BMDCs were cross-linked with 1% formaldehyde for 10 min followed by sonication on ice. The sonicated supernatants were incubated with antibodies against RBPJ overnight at 4 °C with rotation, and normal rabbit IgG (30000-0-AP; Proteintech, Wuhan, China) was used as a negative control. The immune complexes were then immunoprecipitated with salmon sperm DNA/protein A + G agarose slurry for 1 h and eluted out after washes. The eluted DNA was purified using a PCR Purification Kit (Qiagen, Redwood, CA, USA) and subjected to PCR analysis. Primer sequences used in the ChIP assay are listed in Supplementary Table 2.

### Dual-luciferase reporter assay

The *Ciita* promoter regions flanking the putative RBPJ binding sites were amplified from mouse genomic DNA and sub-cloned into a pGL-3 vector. HEK293T cells were co-transfected with luciferase reporter plasmids and a control renilla luciferase reporter vector (20:1). Forty-eight hours post transfection, the relative luciferase activities were accessed using a Dual-Luciferase Reporter Assay System Kit (Promega, Madison, WI, USA) according to the manufacturer’s instructions.

### Statistical analysis

The sample sizes were empirically determined by consulting relevant studies (including animal studies). Exclusion criteria were pre-established before the experiments. If an animal was in an abnormal state (such as death), the corresponding data were excluded. All in vitro studies were conducted with at least 3 independent replications. Data were expressed as mean ± SEM, and their comparisons were accomplished using the Student’s *t* test or one-way ANOVA where applicable. All statistical analyses were carried out using the GraphPad Prism 5 software (GraphPad Software Inc., San Diego, CA). In all cases, *p* values of less than 0.05 were considered as statistically significant.

## Supplementary information

Supplementary Information

unprocessed images of gels and western blots

aj-checklist

## Data Availability

All data that support the findings of this study are present in the paper and/or Supplementary Information. Additional data related to this paper are available from the corresponding authors upon reasonable request.
