# Correction: Ubc9 regulates the expression of MHC II in dendritic cells to enhance DSS-induced colitis by mediating RBPJ SUMOylation

**DOI:** 10.1038/s41419-025-08382-6

**Published:** 2026-01-28

**Authors:** Jing Zhang, Longmin Chen, Qianqian Xu, Yuan Zou, Fei Sun, Qing Zhou, Xi Luo, Yang Li, Cai Chen, Shu Zhang, Fei Xiong, Ping Yang, Shiwei Liu, Cong-Yi Wang

**Affiliations:** 1 Department of Respiratory and Critical Care Medicine, the Center for Biomedical Research, NHC Key Laboratory of Respiratory Diseases, Tongji Hospital Research Building, Tongji Hospital, Tongji Medical College, Huazhong University of Science and Technology, Wuhan, China; 2 Department of Rheumatology and Immunology, the Central Hospital of Wuhan, Tongji Medical College, Huazhong University of Science and Technology, Wuhan, China; 3 Department of Endocrinology, the Central Hospital of Wuhan, Tongji Medical College, Huazhong University of Science and Technology, Wuhan, China; 4 Shanxi Bethune Hospital, Shanxi Academy of Medical Science, Tongji Shanxi Hospital, Third Hospital of Shanxi Medical University, the Key Laboratory of Endocrine and Metabolic Diseases of Shanxi Province, Taiyuan, China

Correction to: *Cell Death & Disease* 10.1038/s41419-023-06266-1, published online 13 November 2023

In the original publication, error was found in Fig 4D. During the assembling of figure, we mistakenly put the same FACS plot from spleen into the mesenteric lymph node (MLN) group. We have confirmed that this correction does not alter the result interpretation or conclusions of our study.

Incorrect Fig. 4D
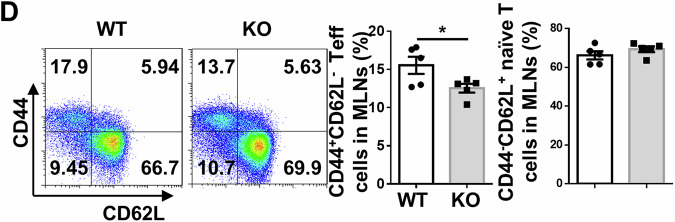


Correct Fig. 4D
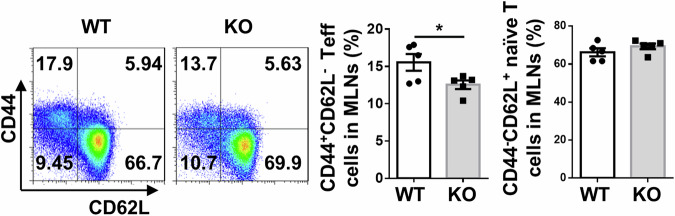


The original article has been corrected.

## Supplementary information

Original data

