# Supplementary material for: Ubc9 regulates the expression of MHC II in dendritic cells to enhance DSS-induced colitis by mediating RBPJ SUMOylation
Source: Cell Death Dis. 2023 Nov 13;14(11):737. doi: 10.1038/s41419-023-06266-1 (PMC10643556; doi:10.1038/s41419-023-06266-1)
Supplement: Supplementary file 1 — Supplementary Information [file 41419_2023_6266_MOESM1_ESM.docx]

**Supplementary Information**

**Ubc9 regulates the expression of MHC II in dendritic cells to enhance DSS-induced colitis by mediating RBPJ SUMOylation**

**Supplementary Figure 1. *Ubc9* deficiency inhibits IL-6 and TNF-α production by T cells in the MLNs.** Single-cell suspension was prepared from mouse MLNs and spleen on day 10 of DSS induction and subject to flow cytometry analysis. n = 5 per group. (A and B) Representative FACS plots and frequencies of TNF-α^+^ (A) and IL-6^+^ (B) cells within the CD3^+^ T cell population in the MLNs. (C and D) Representative FACS plots and percentages of TNF-α^+^ (C) and IL-6^+^ (D) cells gated on CD11c^+^MHC II^+^ DCs in the MLNs. (E and F) Representative FACS plots and frequencies of TNF-α^+^ (E) and IL-6^+^ (F) cells within the splenic CD3^+^ T cells. (G and H) Representative FACS plots and percentages of TNF-α^+^ (G) and IL-6^+^ (H) cells gated on splenic DCs. Data are expressed as mean ± SEM. Statistical difference was determined by unpaired Student’s *t* test. **p* < 0.05.

**Supplementary Table 1. Primer sequences for real-time PCR**

| Gene | Forward (5’-3’) | Reverse (5’-3’) |
| --- | --- | --- |
| *H2-Aa* | TCACATGGCTCAGAAATAGCAAG | CAGGGCACACACCACAGTTT |
| *H2-Ab1* | ACAGCTTATTAGGAATGGGGACT | CACGGTGATGGGACTCTTCA |
| *H2-Eb1* | GAGACACCAGACCACGGTTTA | CTGTCGAAGCACACGTACTC |
| *H2-Eb2* | GTGTGTGGAGTGTGACCAGAT | GGCACGATAATTGTCCAGCAT |
| *H2-Dma* | CTCGAAGCATCTACACCAGTG | TCCGAGAGCCCTATGTTGGG |
| *H2-Dmb2* | GTGTTTCCTTCAACAAAGATCTGCT | ACATCCCAGAAAGGTTGGGTGTG |
| *Cd74* | CCGCCTAGACAAGCTGACC | ACAGGTTTGGCAGATTTCGGA |
| *RFX5* | ACCTGATGCTAAGAGCCCCAA | GTCGTTGTCCGAGAATTTCTGTA |
| *Rfxank* | TCTGCGGAAGGGAAACAACC | AGCGAACTGTCTCAATTTCTCC |
| *Rfxap* | GAAGAAGAGCGACCAGGCAC | CAGGGCGATCACCAAAGGAT |
| *Creb* | AGCCGGGTACTACCATTCTAC | GCAGCTTGAACAACAACTTGG |
| *Nfya* | GTTAATGGTGCAAGTCAGTGGA | TCTGCTGTAAACCTTGTGTTCC |
| *Nfyb* | GCCTCCCAGCTAGGGATTTC | TTCCTGTTTGAGGTATGGCATTT |
| *Nfyc* | GGCAGCCCAGATTTTTATCACT | GGAGGTTTCAGTTCATCTCTTGG |
| *Ciita* | AGACCTGGATCGTCTCGTG | AGTGCATGATTTGAGCGTCTC |

**Supplementary Table 2. Primer sequences for ChIP-qPCR**

| Gene | Forward (5’-3’) | Reverse (5’-3’) |
| --- | --- | --- |
| *Ciita-169* | TTCCCCAAGTGAGCTACAGTTTCC | GTGCCTGATGAACGGAAATGG |
| *Ciita-1956* | GACTCTCATTGGACAGCCCTAGC | TGGTGATGGTGATGGTGATGGT |
