# Supplementary figures and images for: Ubc9 regulates the expression of MHC II in dendritic cells to enhance DSS-induced colitis by mediating RBPJ SUMOylation
Source: Cell Death Dis. 2023 Nov 13;14(11):737. doi: 10.1038/s41419-023-06266-1 (PMC10643556; doi:10.1038/s41419-023-06266-1)

Fig. 1C

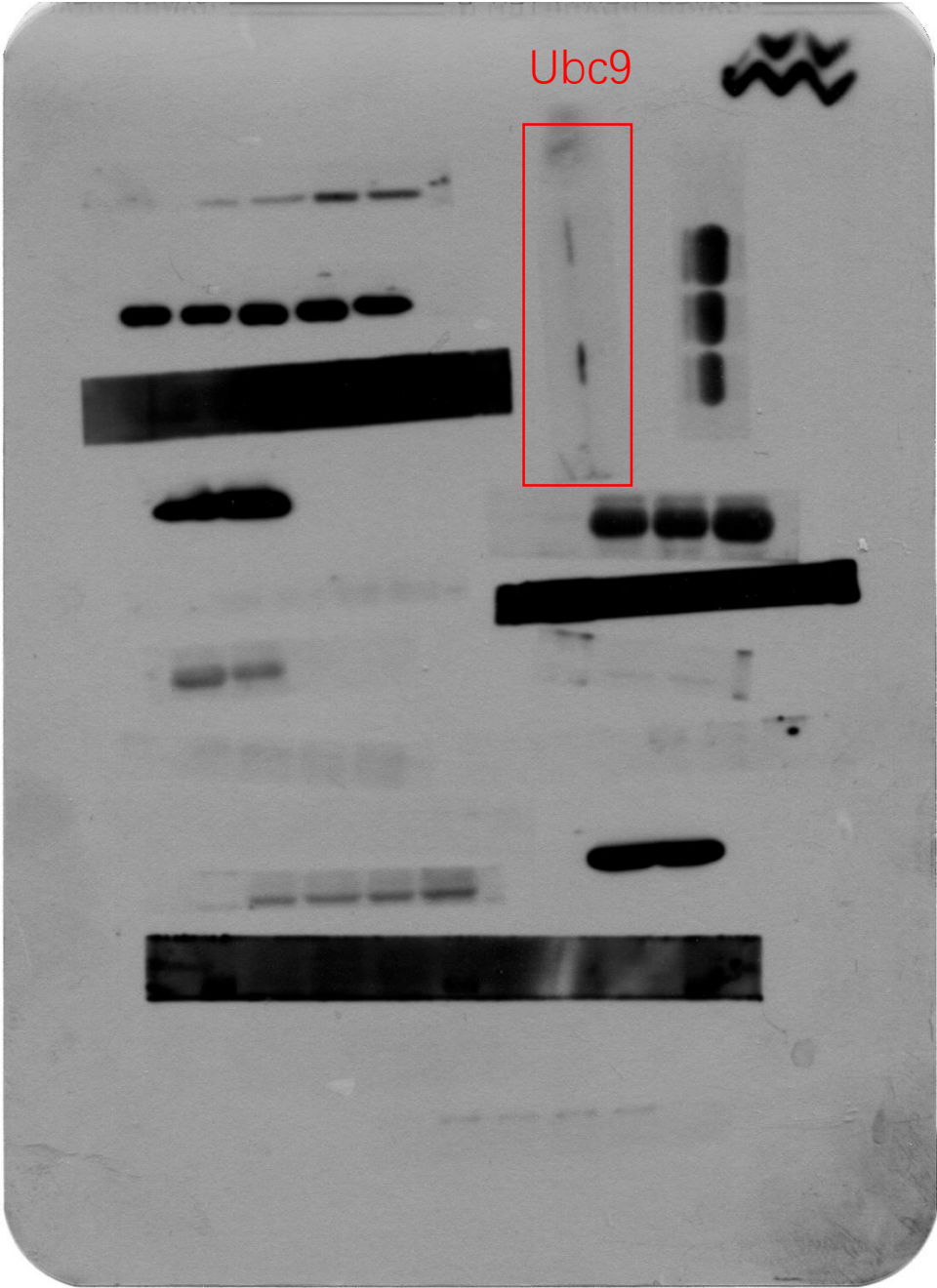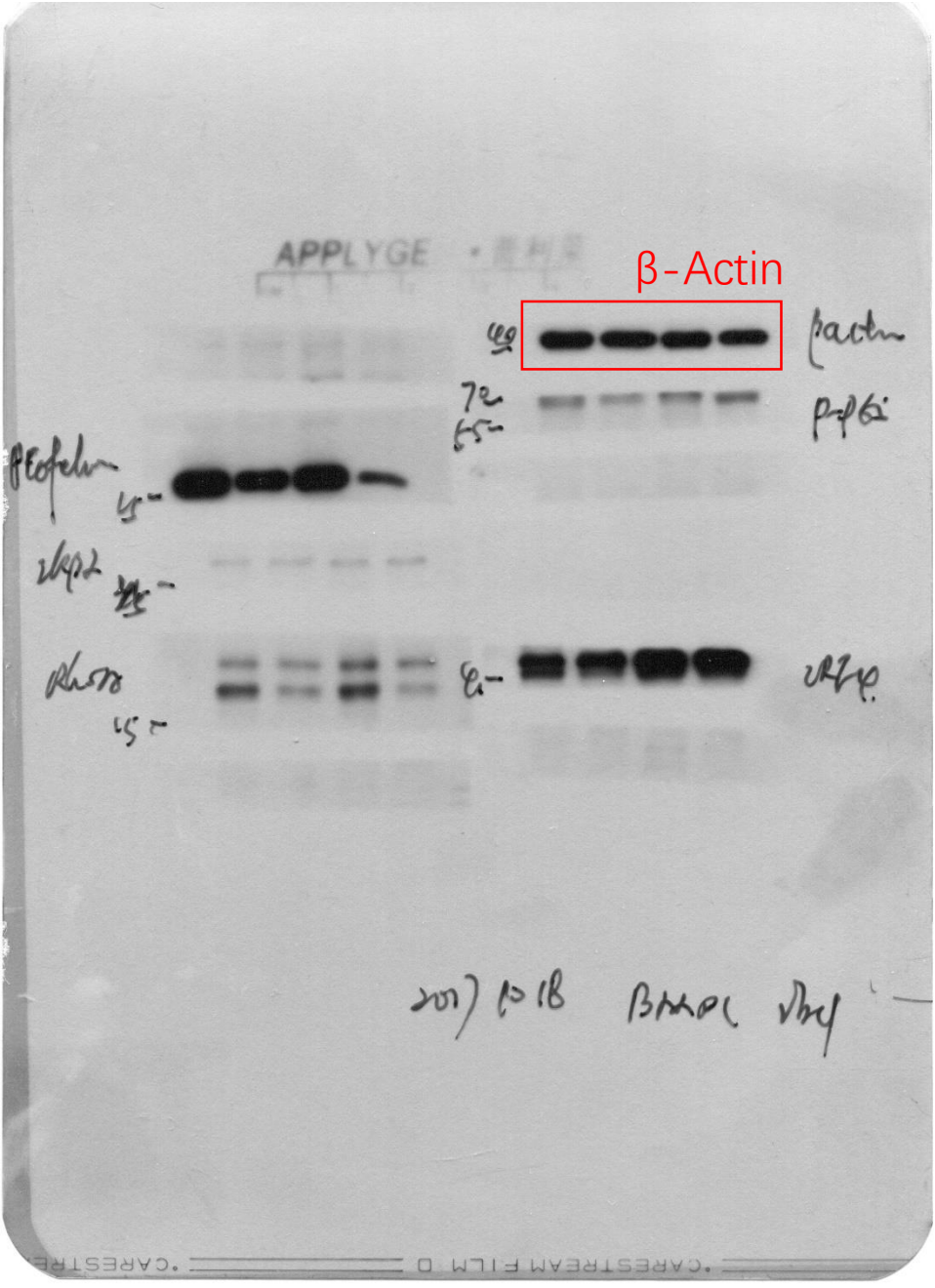

Fig. 1D

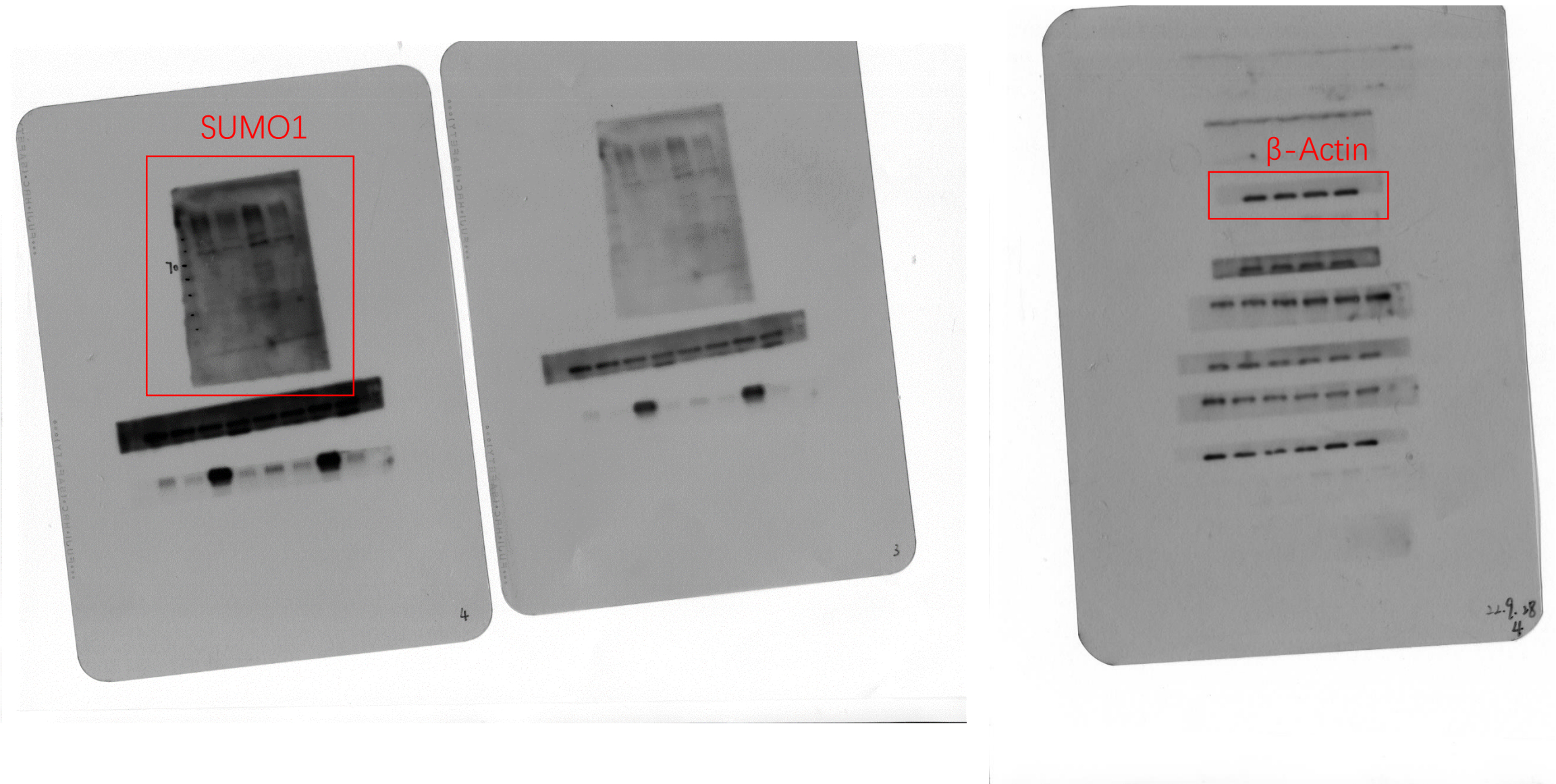

Fig. 5C

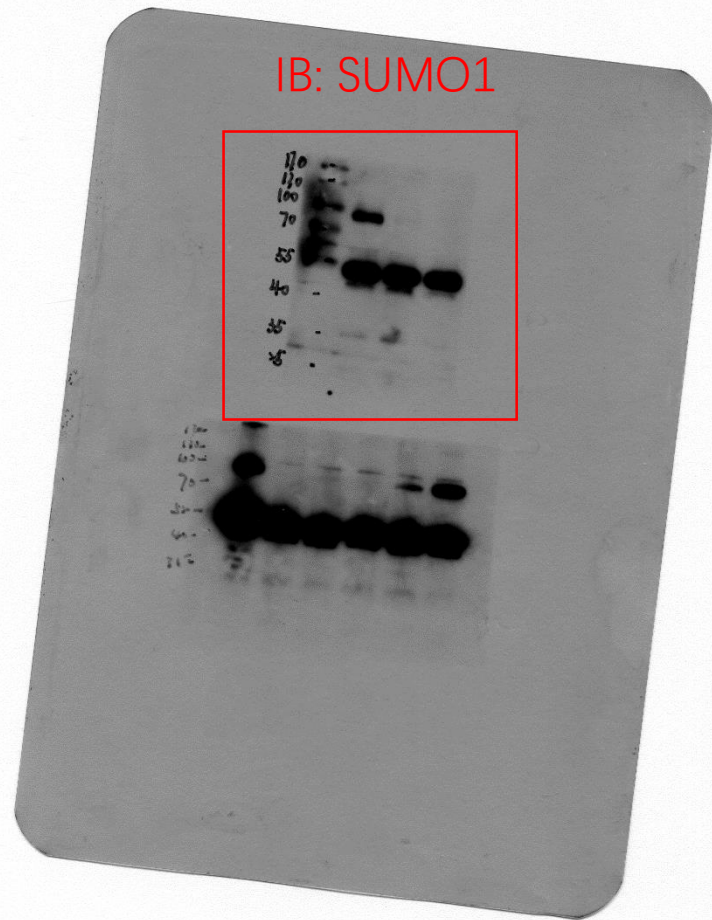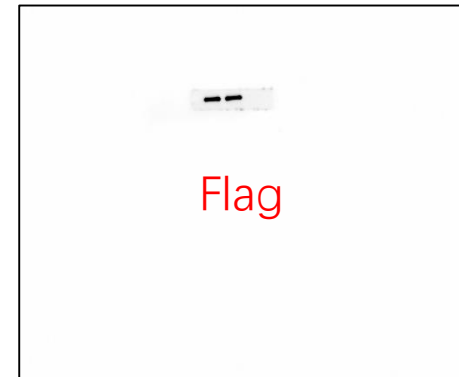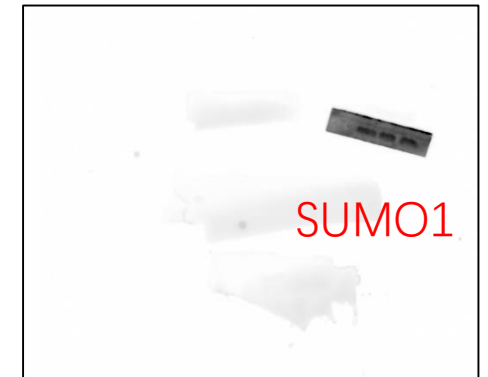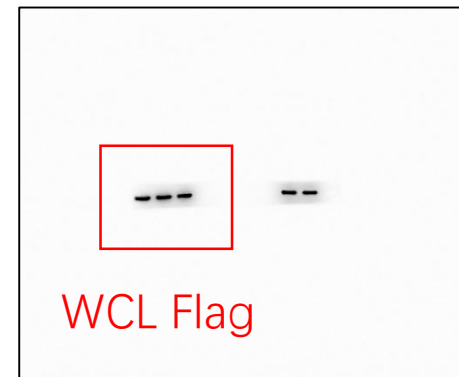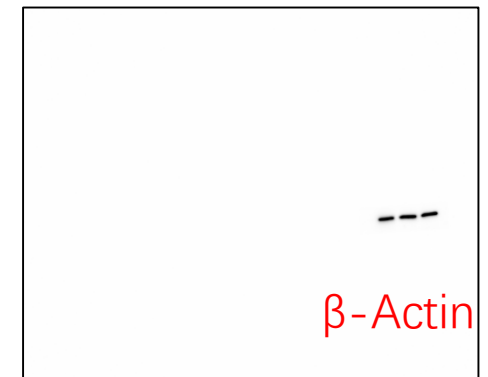

Fig. 5D

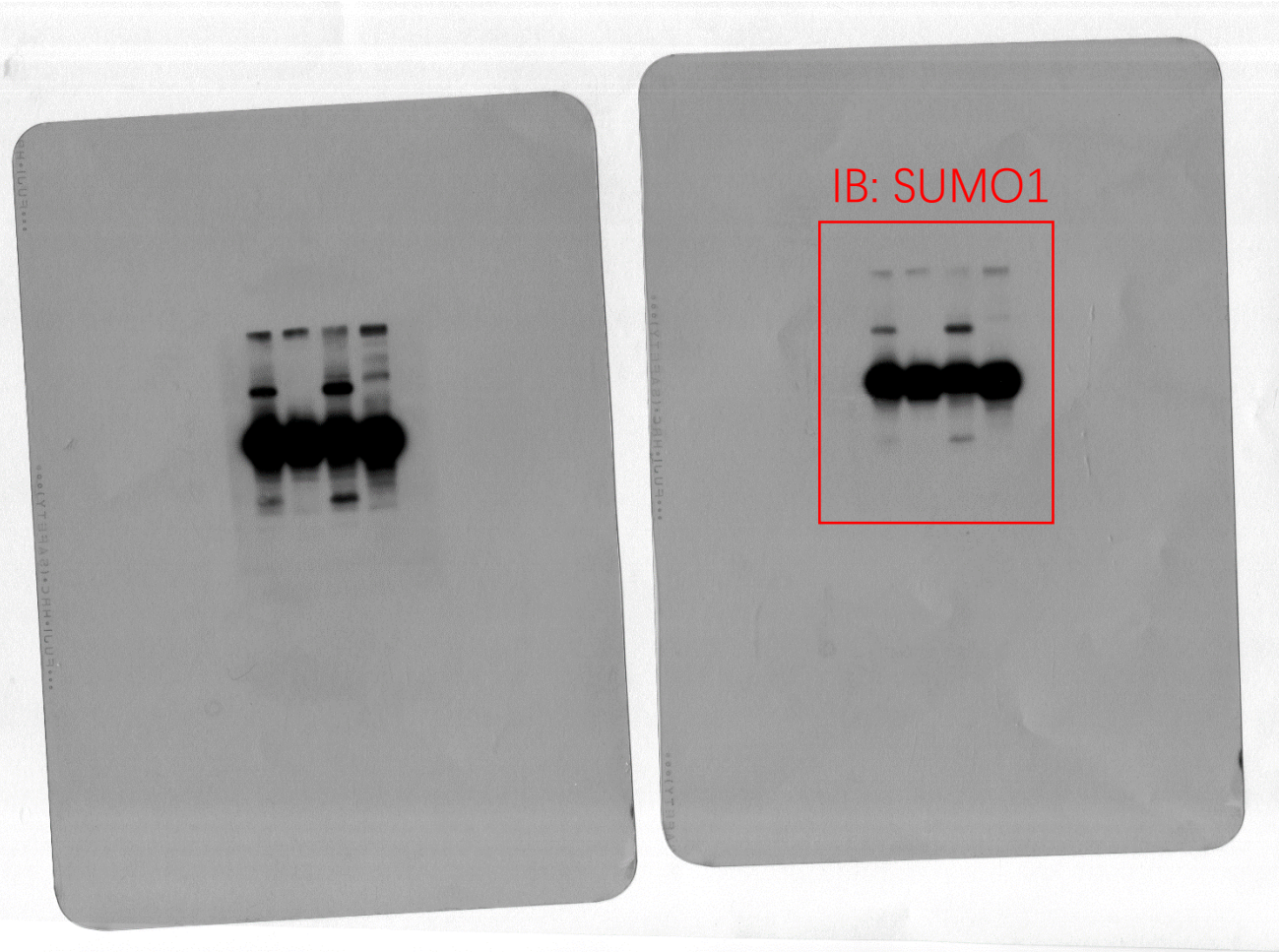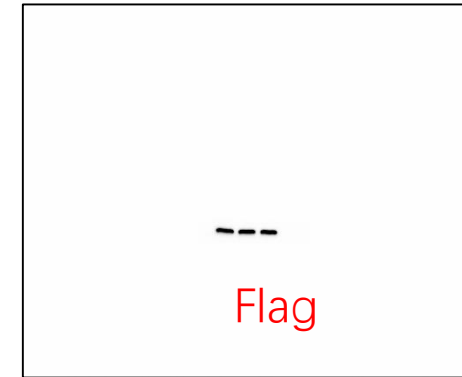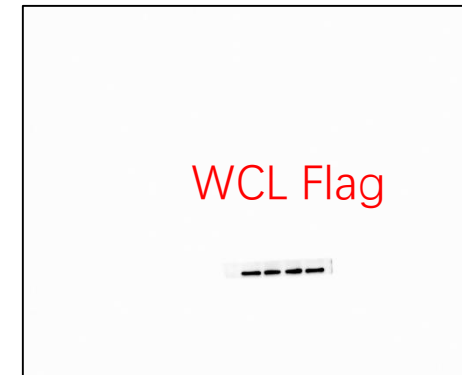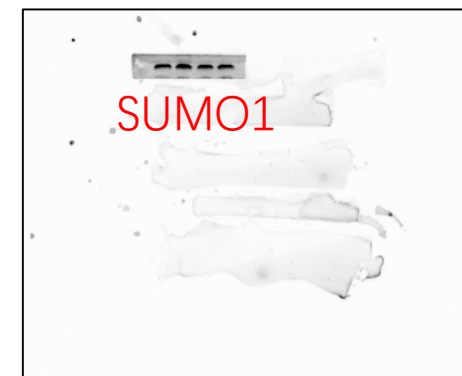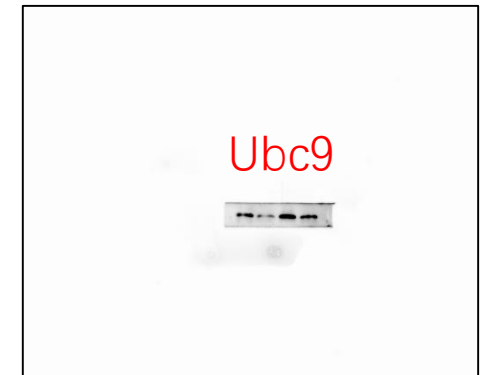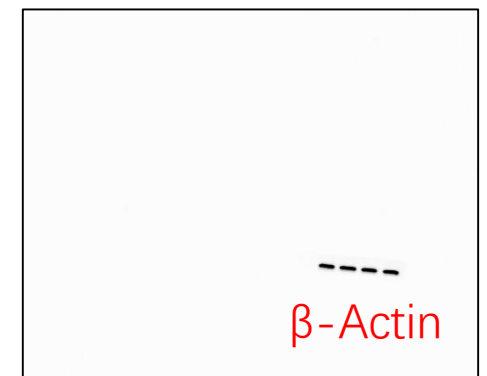

Fig. 5E

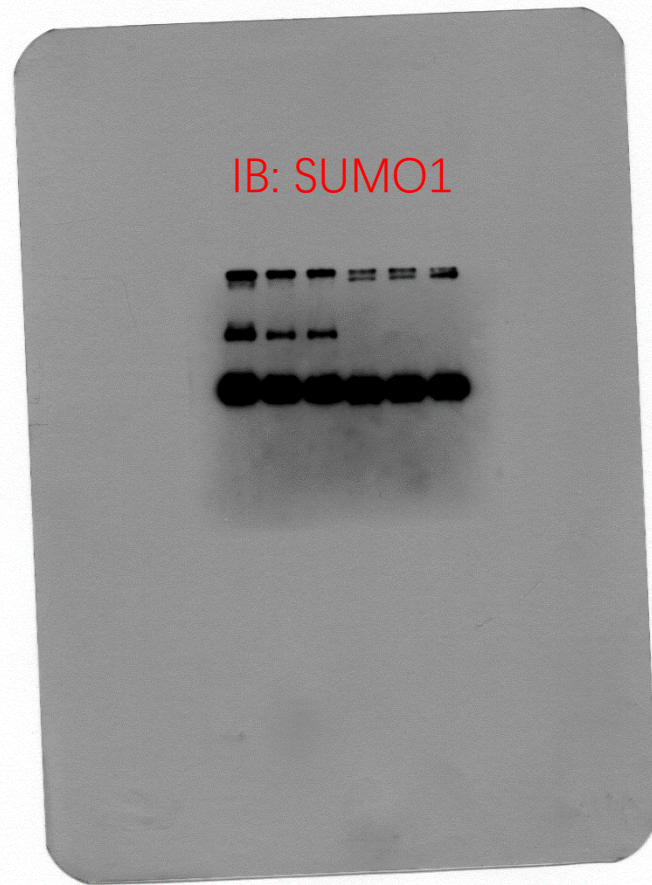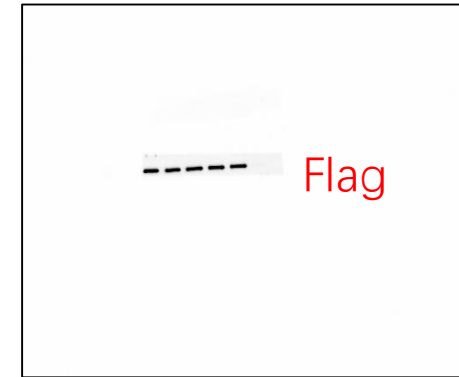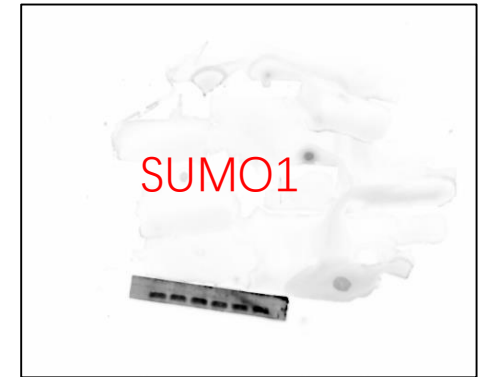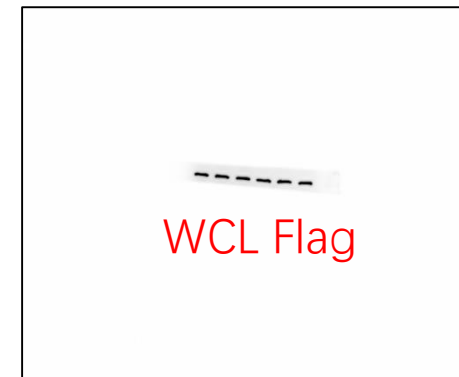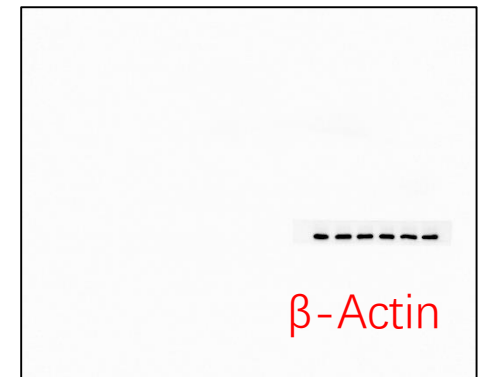

Fig. 5F

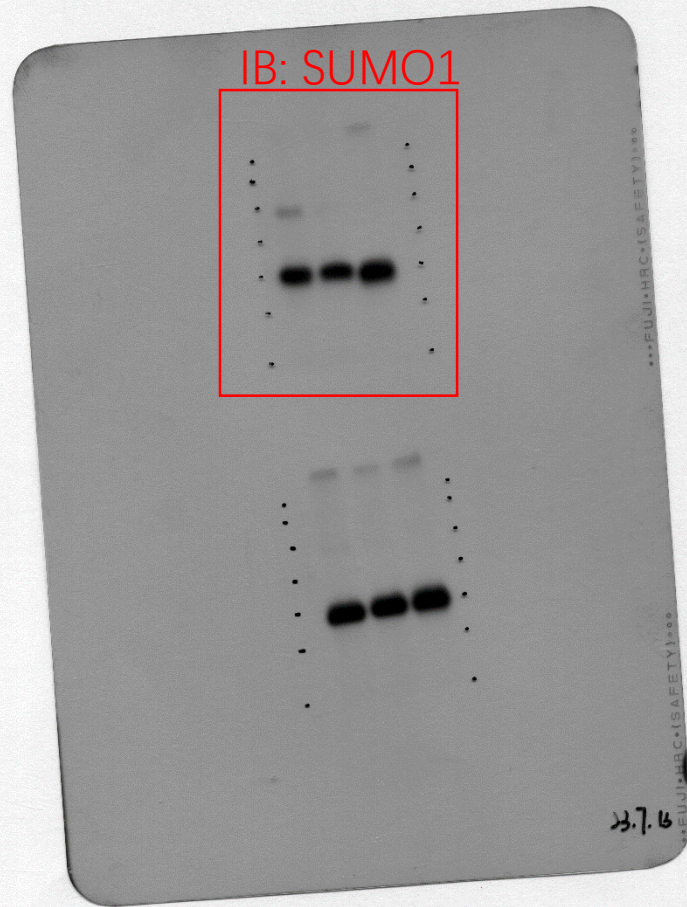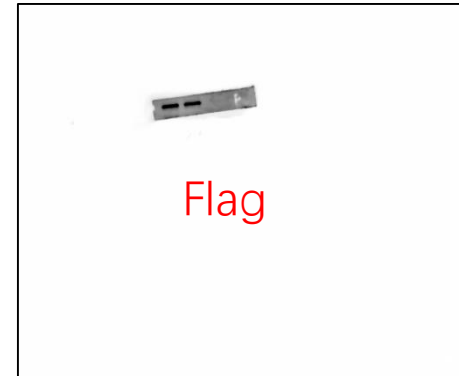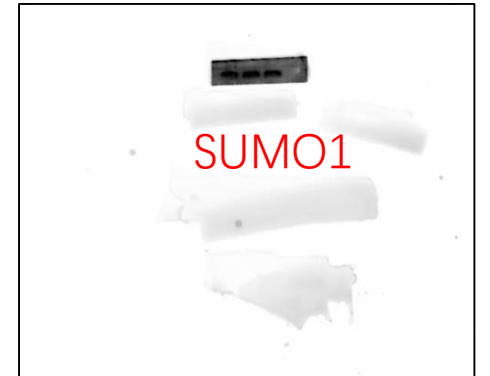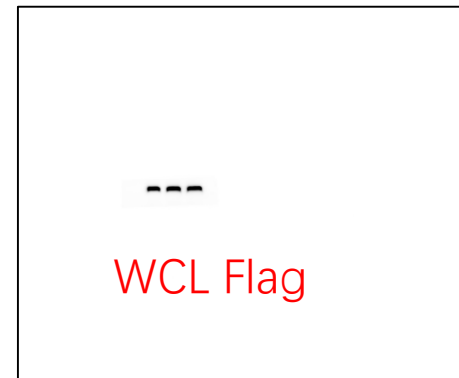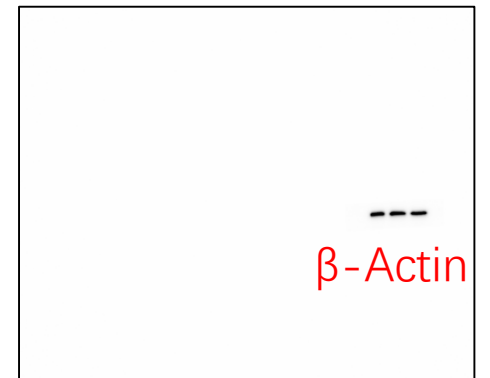

Fig. 6A

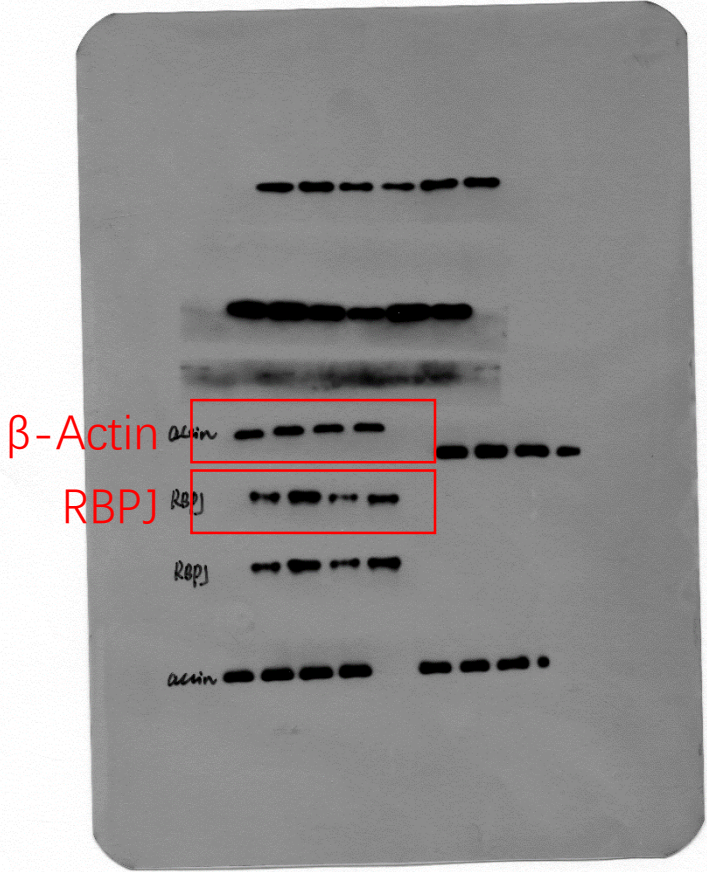

Fig. 6C

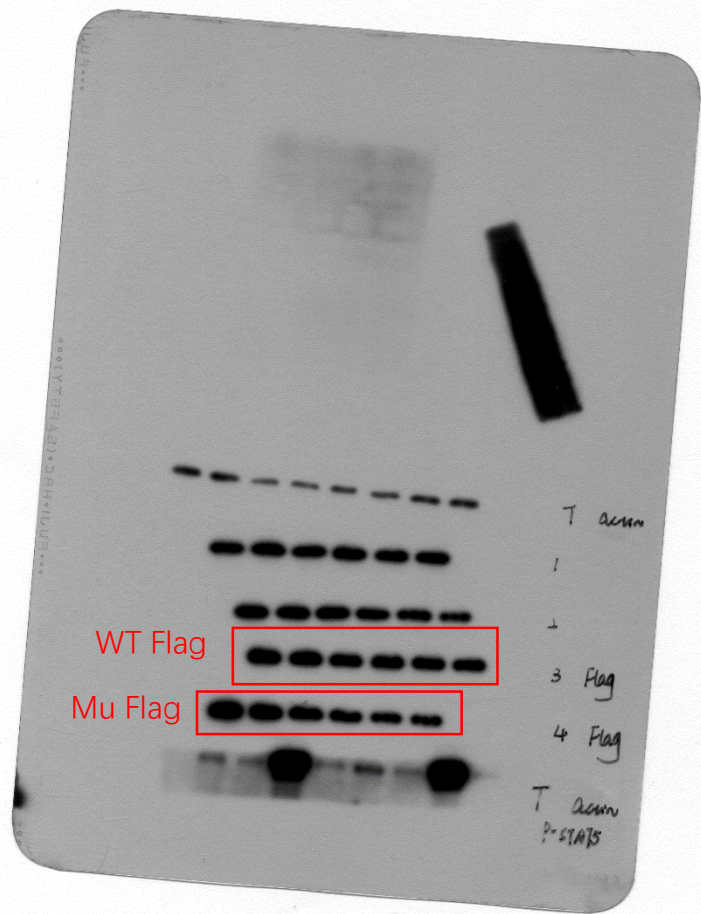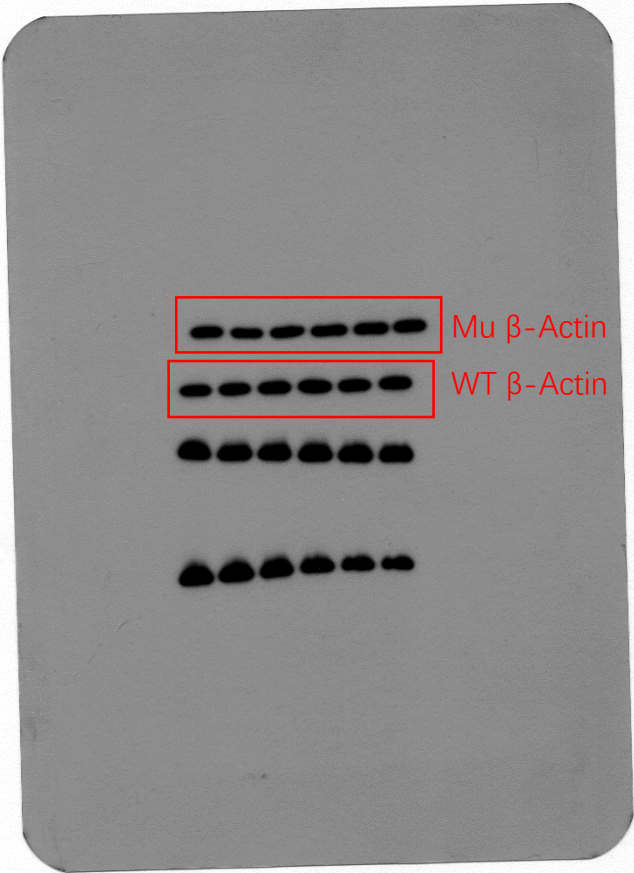

Fig. 6D

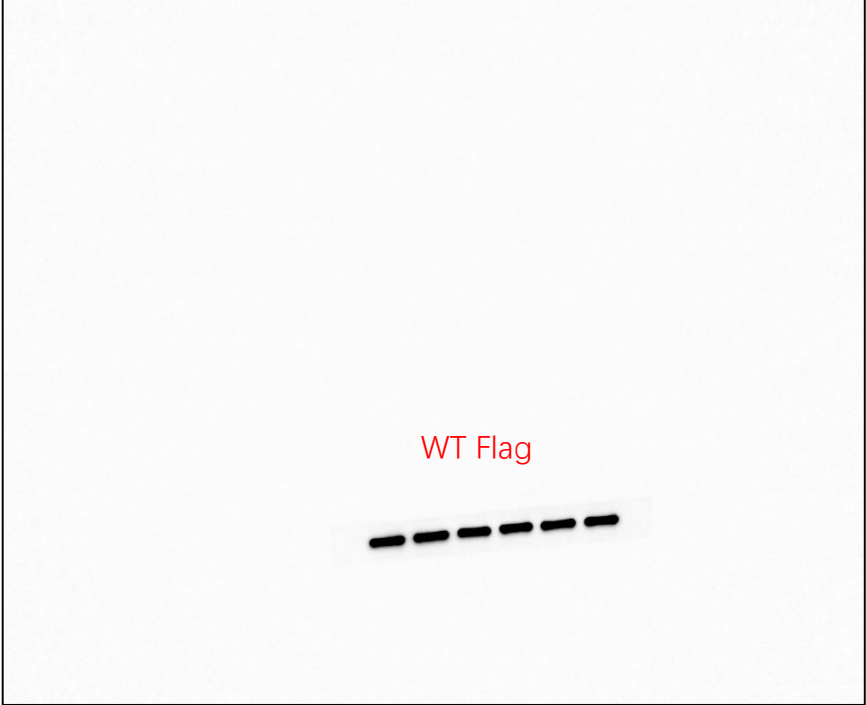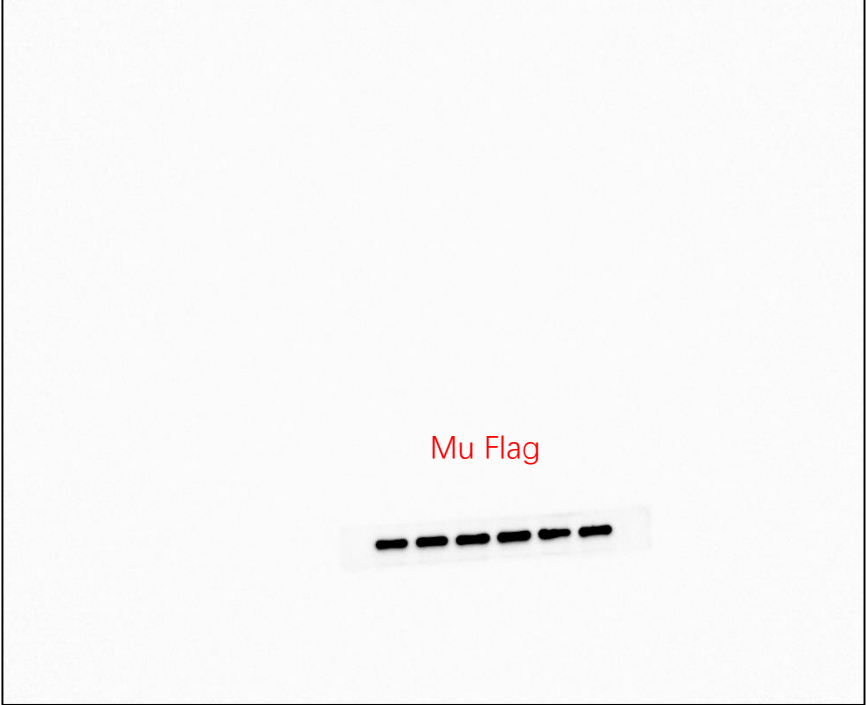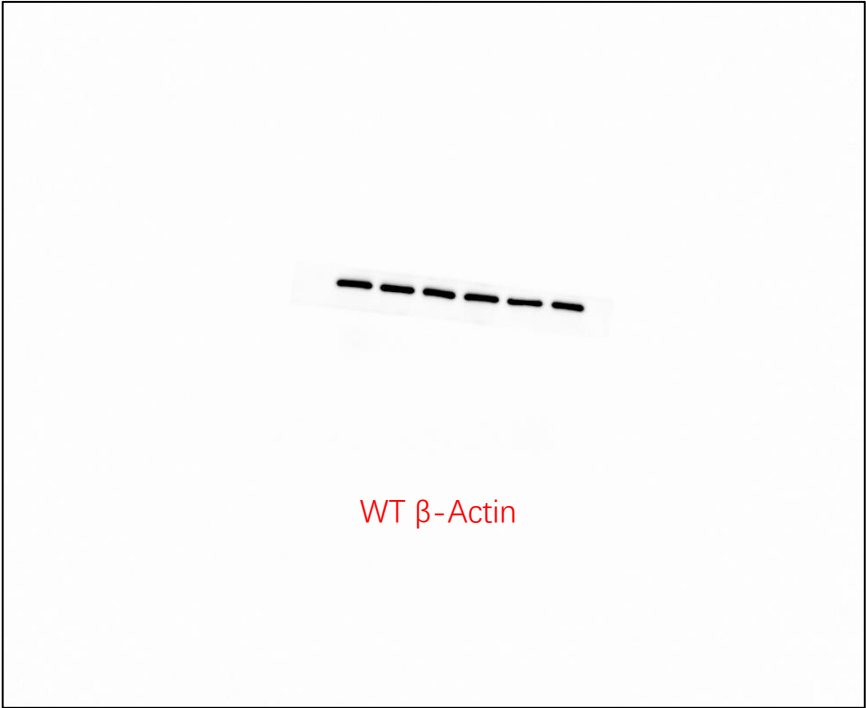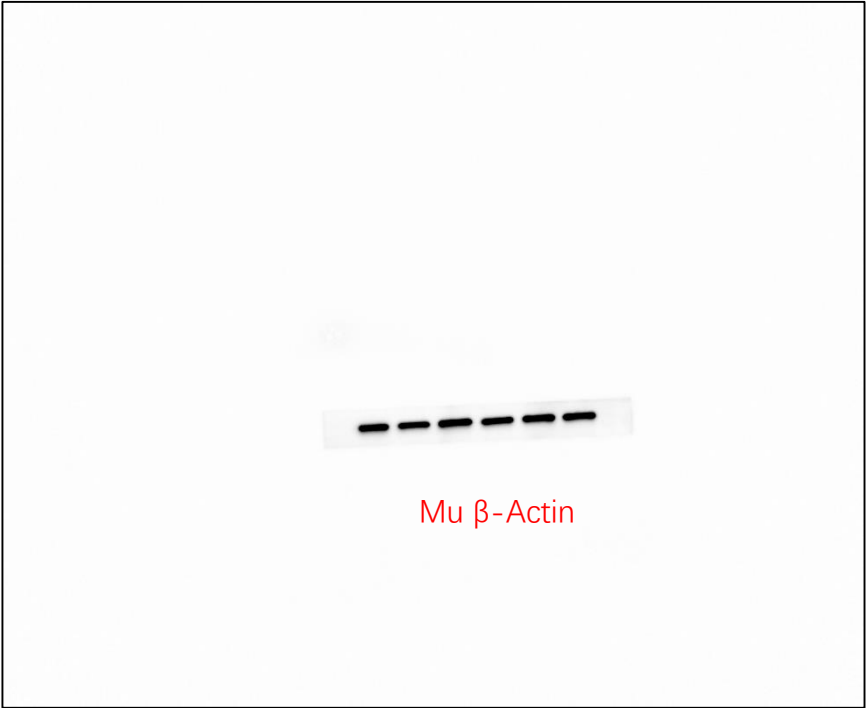

Fig. 6E

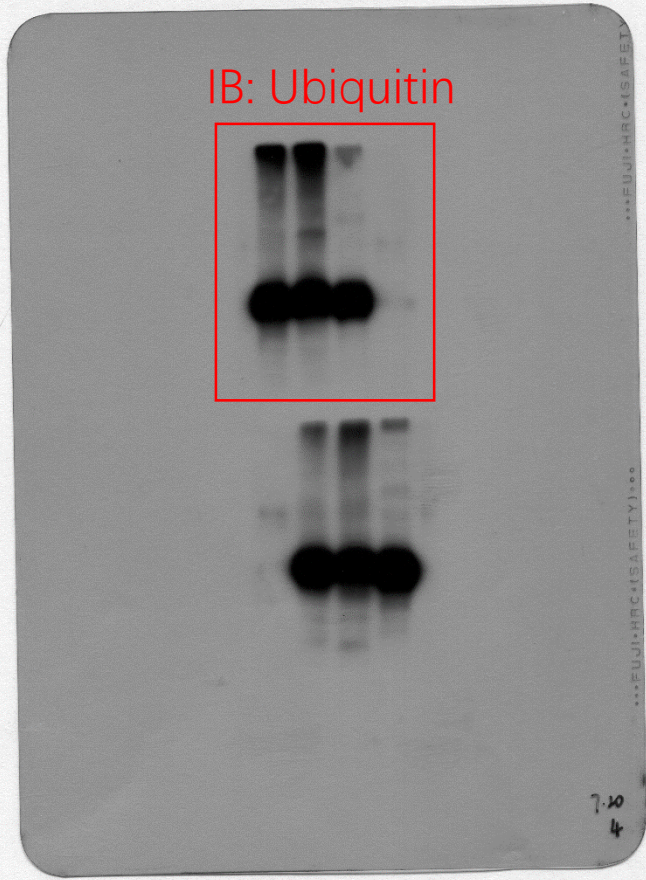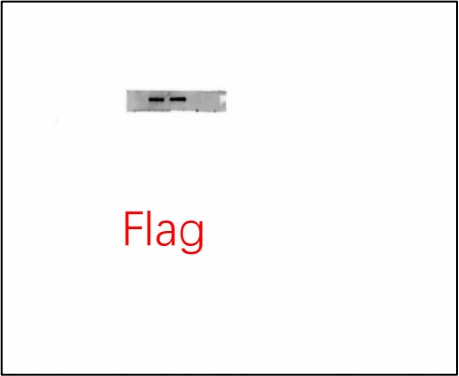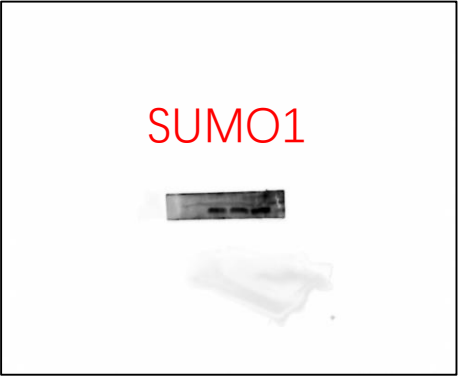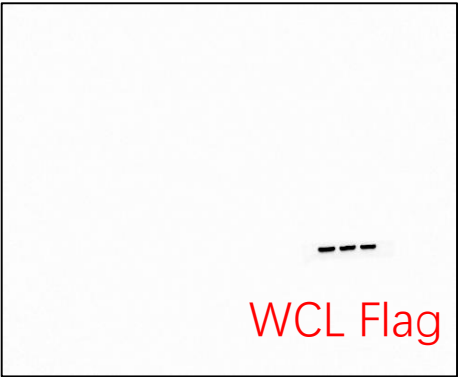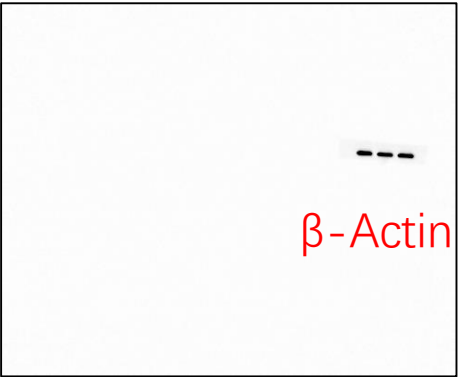

Fig. 6F

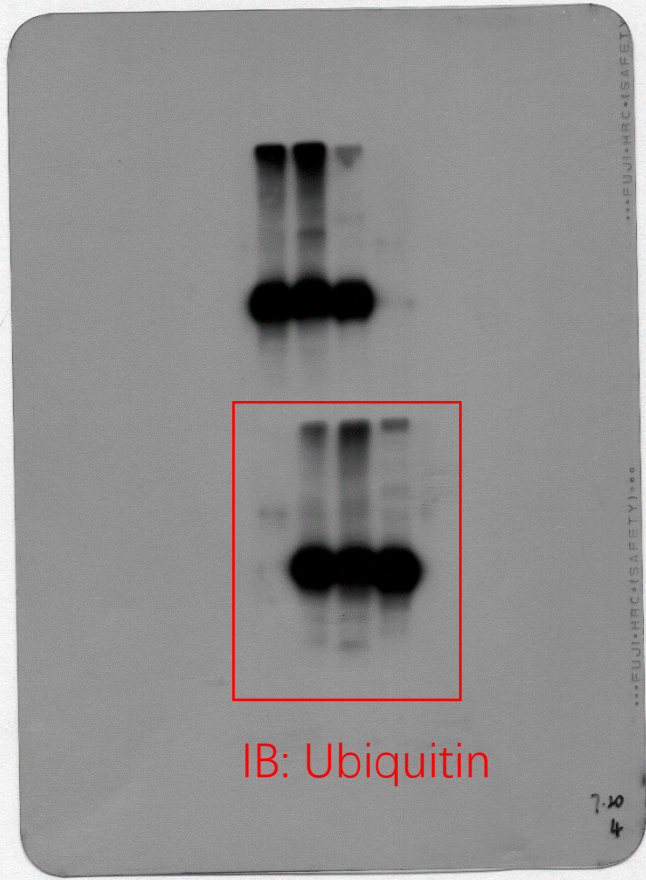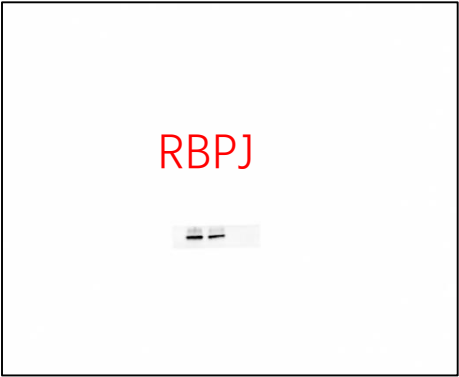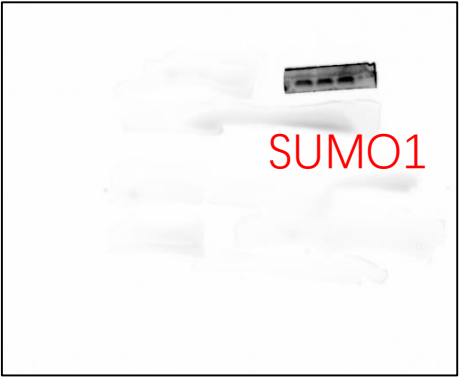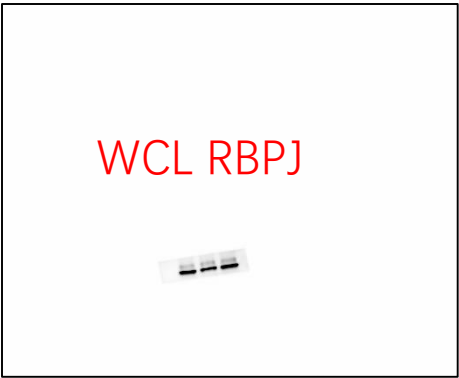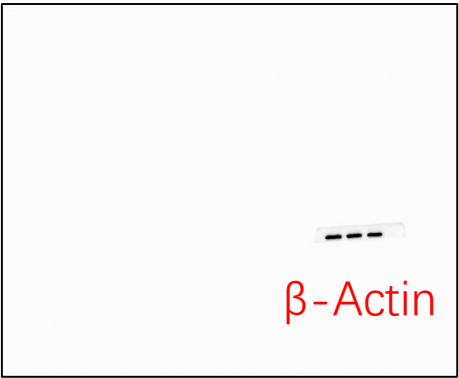

Fig. 6I

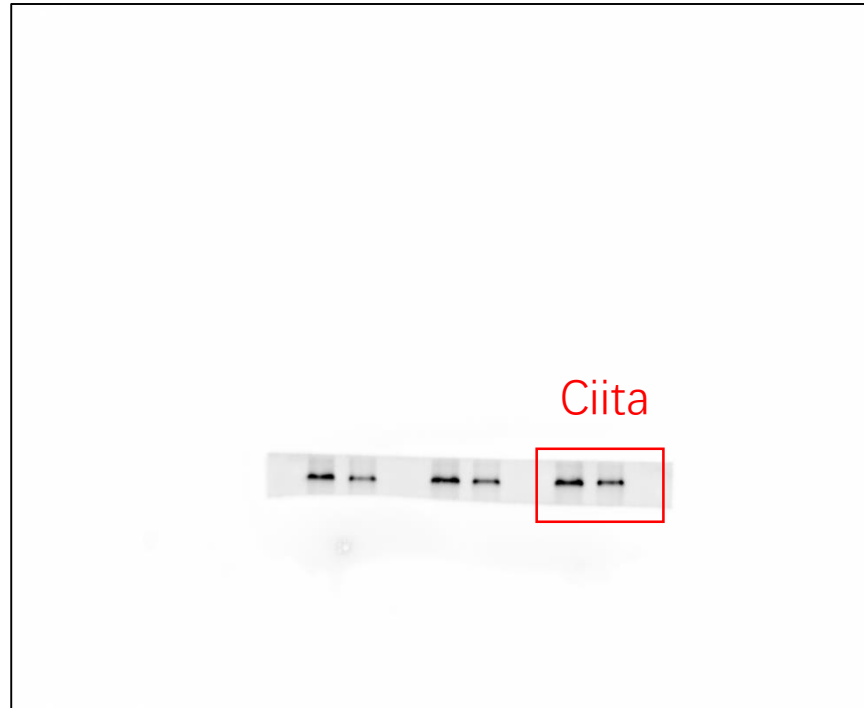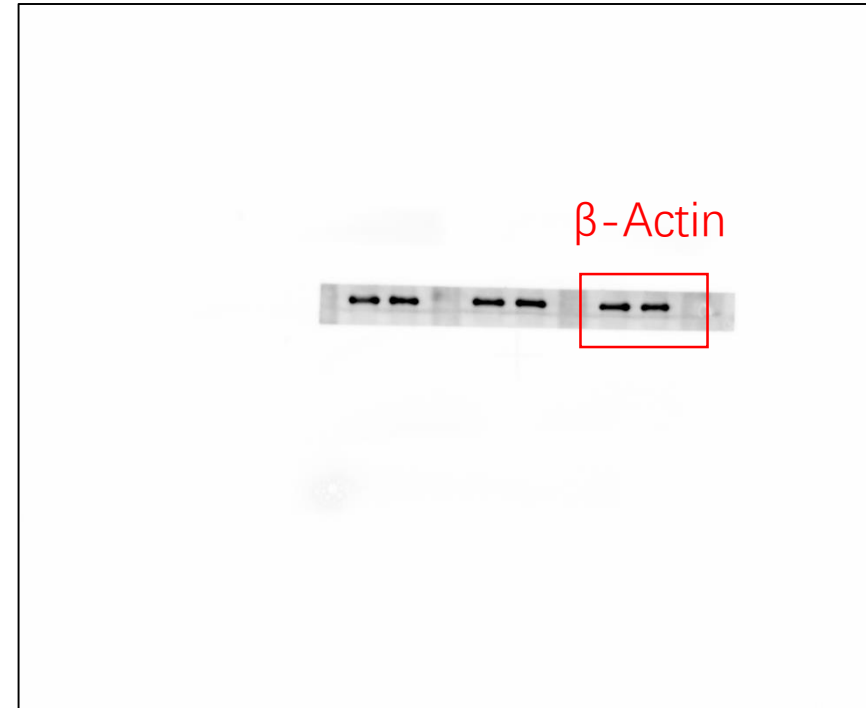

Fig. 1B

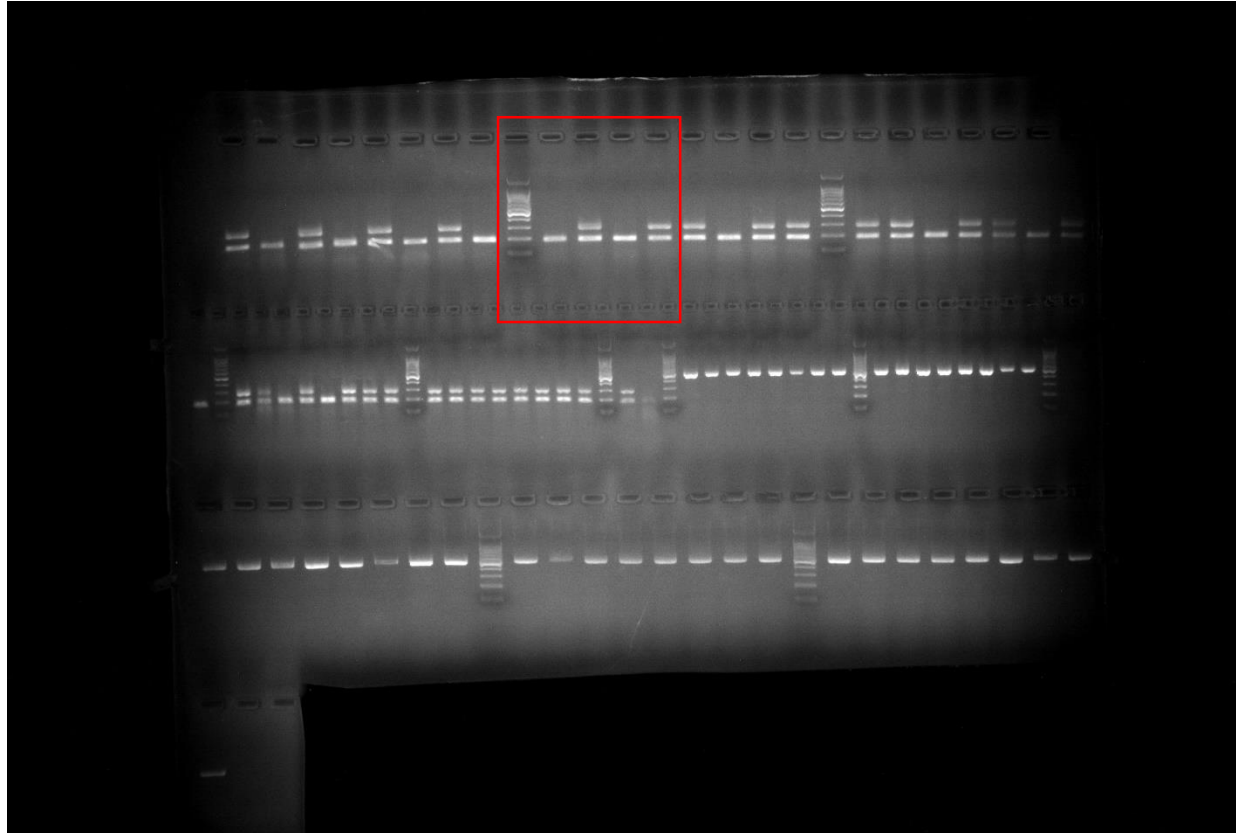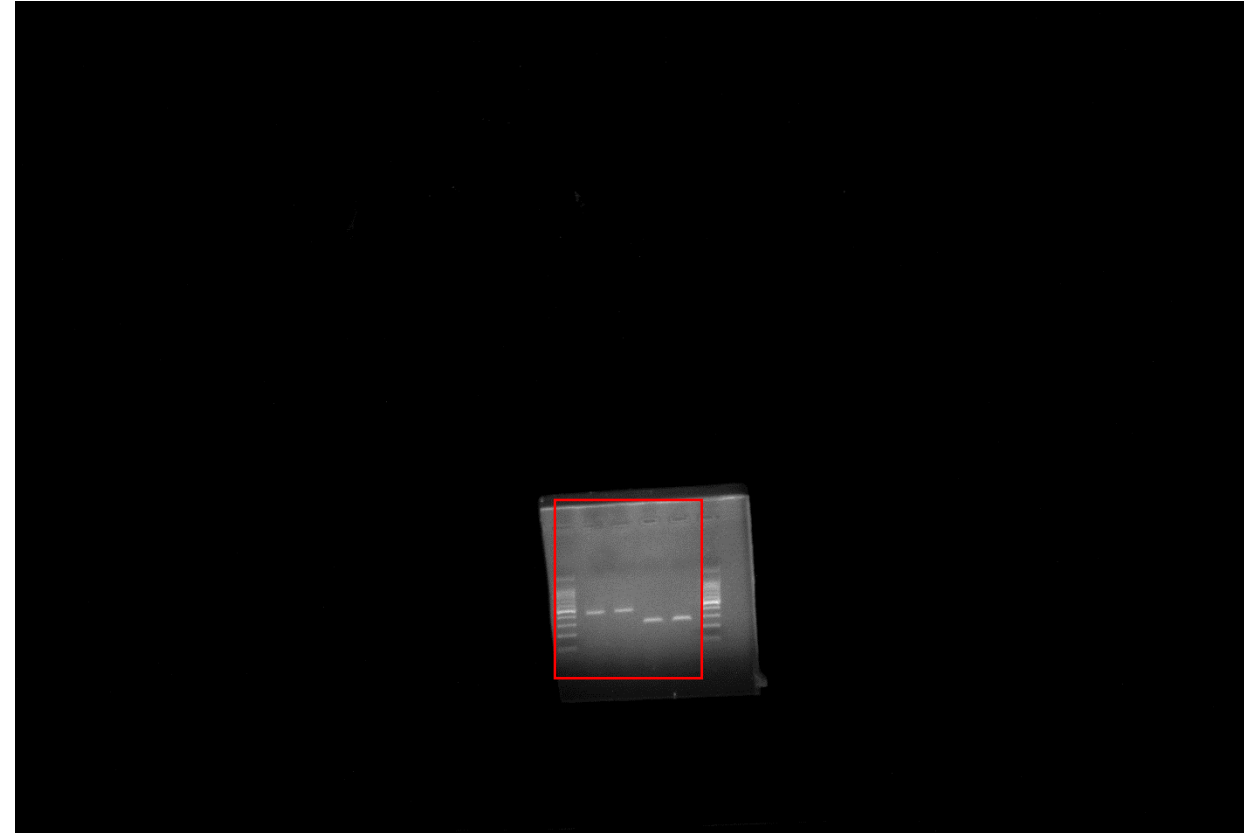

Fig. 6K

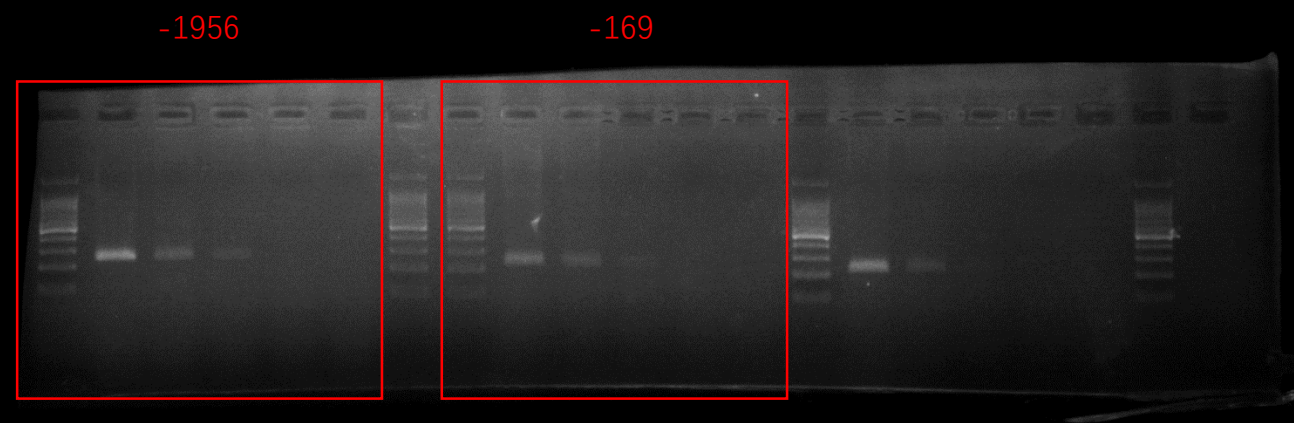

Supplement: Supplementary file 2 — unprocessed images of gels and western blots [file 41419_2023_6266_MOESM2_ESM.pdf]
