# Supplementary figures and images for: Correction: Ubc9 regulates the expression of MHC II in dendritic cells to enhance DSS-induced colitis by mediating RBPJ SUMOylation
Source: Cell Death Dis. 2026 Jan 28;17(1):145. doi: 10.1038/s41419-025-08382-6 (PMC12852650; doi:10.1038/s41419-025-08382-6)

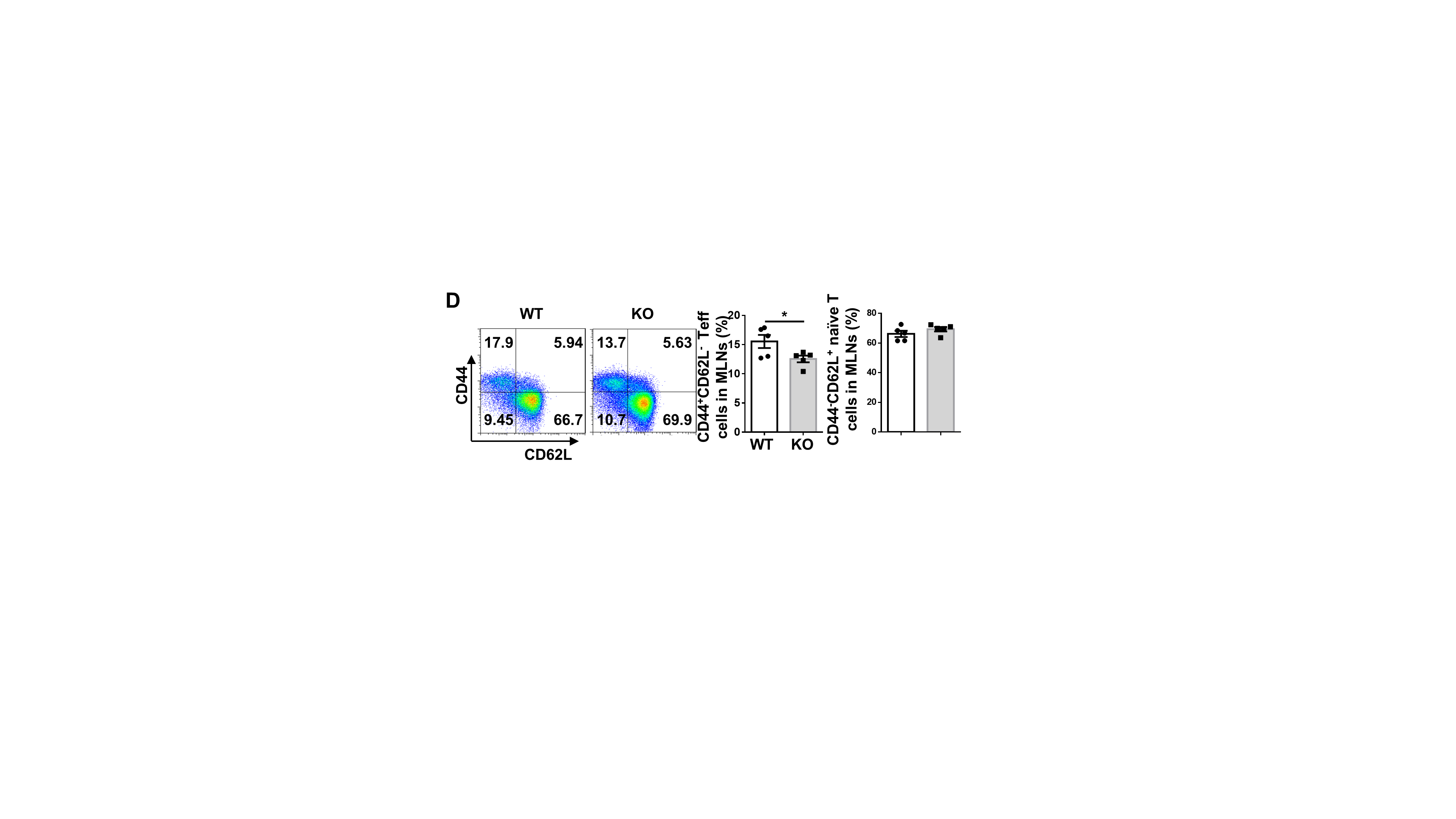

Supplement: Supplementary file 1 — Original data [file 41419_2025_8382_MOESM1_ESM.tif]
